# Clinical Applications of Bovine Colostrum in GastrointestinaI Disorders: Mechanisms, Evidence, and Therapeutic Potential

**DOI:** 10.3390/ijms262110673

**Published:** 2025-11-01

**Authors:** Yusuf Serhat Karakülah, Yalçın Mert Yalçıntaş, Mikhael Bechelany, Sercan Karav

**Affiliations:** 1 Department of Molecular Biology and Genetics, Çanakkale Onsekiz Mart University, Çanakkale 17000, Turkey; yusufserhatkarakulah@gmail.com (Y.S.K.); yalcinmertyalcintas@stu.comu.edu.tr (Y.M.Y.); 2 European Institute for Membranes (IEM)—UMR 5635, University of Montpellier, ENSCM, CNRS, 34090 Montpellier, France

**Keywords:** colostrum, gastrointestinal, lactoferrin, therapeutic, clinical

## Abstract

Bovine colostrum stands out as a natural supplement with rich bioactive components that attract attention for its therapeutic potential in the maintenance and improvement of gastrointestinal (GI) health. The major bioactive components of bovine colostrum include immunoglobulin (Ig) (especially immunoglobulin G), lactoferrin (LF), growth Factors (IGF-I, TGF-β, EGF), oligosaccharides (OS), and bioactive peptides. These components play a role in epithelial repair, suppression of inflammation, balancing the microbiota, and enhancing the mucosal barrier. Various animal models and recent human studies show that bovine colostrum has various positive effects against gastrointestinal tract diseases such as inflammatory bowel disease (IBD), irritable bowel syndrome (IBS), non-steroidal anti-Inflammatory drug (NSAID)-induced enteropathy, and necrotizing enterocolitis (NEC). These effects include preservation of epithelial integrity, reduction of inflammatory markers, and improvement of intestinal permeability. Studies on the tolerability and efficacy profiles of various bovine colostrum formulations for oral, oropharyngeal, and enteral administration are increasing. In this review, the multifaceted effects of bovine colostrum on the gastrointestinal tract are explained at a mechanistic level, and potential areas of study for clinical translation are presented. Bovine Colostrum stands out as a promising natural biotherapeutic agent for both preventive and therapeutic approaches.

## 1. Introduction

Colostrum is a distinct form of milk secreted by mammals during the first 24–72 h after parturition. Colostrum is an essential source of nutrients for the growth and development of newborn individuals. Compared to mature milk, colostrum has significantly more beneficial nutritional composition and a greater concentration of bioactive compounds [1].

The recent studies about the Bovine colostrum (BC) proved that BC has a rich nutrient composition and immune supportive, regulatory, and growth factor bioactive components such as immunoglobulins (IgG is the highest in BC), LF, lysozyme, insulin-like growth factors (IGF), and glycans (oligosaccharides, glycoproteins, glycolipids). Owing to these components, BC provides significant positive effects in terms of regulation of the immune system, enhancement of the gastrointestinal (GI) system, supporting the growth and repair mechanisms, antibacterial and anti-inflammatory effects [2]. Research demonstrates that BC supplementation to children aged 4–30 months results in remarkable beneficial effects on duration of diarrhea and stool frequency [3]. Moreover, BCs positive effects highlighted the beneficial role of BC in reducing bacterial translocation across the intestinal barrier, particularly in individuals undergoing abdominal surgery [4]. A study conducted on mice demonstrates that the BC supplementation reduces the production of IL-6 and increases the production of IL-10. These effects indicate that the suppression of IL-6 and the increase of IL-10 show the anti-inflammatory and immunomodulatory effects of BC [5]. Furthermore, BC has remarkable beneficial effects on treatment and prevention of GI diseases such as infectious diarrhea, inflammatory bowel disease (IBD), irritable bowel syndrome (IBS, NSAID-Induced enteropathy, and necrotizing enterocolitis (NEC) in preterm infants.

BC modulates intestinal permeability because of its composition that is rich in bioactive components and has selective positive effects on microbiota. BC shows favorable effects on the prevention of adverse effects on GI tract. Moreover, BC plays a role in strengthening the digestive system via its prebiotic properties, and BC’s inflammation-reducing effects occur due to suppressing the NF-kB (nuclear factor kappa B) with its bioactive compounds. This effect of the BC occurs mainly in the epithelial cells of the GI tract [6]. Additionally, BC mitigates the symptoms of digestive diseases such as diarrhea and virus-induced secondary ulcers and lowers the intestinal permeability. BC may also prevent the gastric lesions occurring due to the consumption of non-steroidal anti-inflammatory drugs when it’s consumed with these NSAIDs [7].

BC also has effects on intestinal epithelial cells that support mucosal integrity and enhances tissue repair; these repairing effects can be determined as DNA and RNA repair, and BC shows anti-inflammatory effects by suppressing the NF-kB(a transcription factor that plays a critical role in immune system and inflammation processes) in intestinal epithelial cells [6]. These repairing effects of BC become faster by increasing cell proliferation. Moreover, the GF s in BC’s rich bioactive composition support tissue repairing mechanisms. The epidermal growth factor (EGF) is one of these GFs. EGF contributes significantly to the development of the intestinal mucosa in newborn individuals and in the re-epithelialization of the intestinal epithelial cells after tissue damage. EGFs in BC are vital because of these features [8]. LFs are also critical for the proliferation and differentiation of epithelial cells. In a study that was conducted on mice, it was established that LFs enhance tissue repair in intestinal epithelial cells [9].

BC has immunoregulatory effects, and these effects have diverse mechanisms. It appears that bioactive components such as Igs, GFs, OS, LFs in BC have immunomodulatory effects that prevent GI infections [10]. Moreover, the immunomodulatory effect of BC can be demonstrated by the transfer of maternal Igs to cattle via colostrum because the passive immunity cannot be transferred through the placenta in cattle. The involvement of LF in iron absorption may promote various immune responses; thus, it is assumed that LF-containing supplements can be used for inducing immunomodulatory responses [11]. BC supplementation can regulate the functions of the cell subsets that are crucial for the immune system, such as lymphocytes, macrophages, and dendritic cells, and these regulatory effects have been observed in studies. IgA (a major antibody of mucosal immunity) is reduced, and susceptibility to respiratory tract diseases increases after intense exercise in athletes. This increased state of susceptibility is prevented by BC supplementation in athletes. With BC supplementation for 2–12 weeks, an increase in IgA has been observed in saliva. BC has beneficial immunomodulatory effects on older adults, newborns, and colorectal cancer patients [12]. Additionally, LF s in the BC affect the immune system by inducing apoptotic activity in cells, elevating the activity of cytotoxic T cells and natural killer (NK) cells [10].

BC also has multifaceted effects on the microbiota. These effects can be diversified by increasing beneficial bacteria, having a barrier effect against pathogens, maintaining microbial balance, and increasing intestinal permeability. Various studies have been conducted on the effects of BC on microbiota. In a study conducted on a mouse model in which the effect of BC on microbiota was observed, it was revealed that BC was associated with the taxonomic abundance of beneficial bacterial species. It was also found that BC administration was associated with a decrease in some proinflammatory bacterial species [13]. In a study with premature pigs, it was observed that the addition of BC to formula food altered the gut microbiota and improved gut function. BC treatments have a wide range of beneficial effects on the gut microbiota, as supported by some animal studies. Although previous reviews have evaluated the nutritional value and immunological properties of BC, most studies have focused more on compositional aspects and general GI benefits. In contrast, this review distinguishes itself from previous studies by systematically integrating current mechanistic and clinical evidence regarding the therapeutic effects of BC in GI diseases. Furthermore, by linking molecular mechanisms to translational outcomes, it aims to provide a comprehensive framework for future clinical applications. In this review, we have discussed and evaluated the bioactive components and clinical applications of BC for GI health. Action mechanisms in the GI tract, preclinical and clinical studies, formulations, and administration routes, regulatory and safety considerations of BC are discussed, and future directions are determined.

## 2. Key Bioactive Components Relevant to GI Health

The bioactive components of BC are determined by standardized chemical, chromatographic, and immunochemical methods. Protein and fat contents of BC were measured by the Kjeldahl method (AOAC 991.20) and VDLUFA VI C15.2.1 procedures, respectively, and analyzed by high-performance liquid chromatography (HPLC) using an Aminex HPX 87H column [14]. Concentrations of major Igs (IgG, IgA, IgM) were determined by radial immunodiffusion (RID) and ELISA methods to obtain an accurate immunological profile [14,15]. Under field conditions, the total solids amount, which reflects the general quality of colostrum, is evaluated by the Brix refractometry method, which shows a high correlation with protein and IgG levels [15]. In addition, recent systematic reviews show that chromatographic, spectroscopic, and immunochemical techniques are still used as the main methods in the analysis of chemical and bioactive components of bovine colostrum [11].

### 2.1. Lactoferrin

LF s are multifunctional glycoproteins in BC. The key characteristics of LFs are that they are iron-binding proteins. These iron-binding features of LFs provide them with bactericidal effects. In addition to this antimicrobial activity, Lfs also shows immunomodulator effects (as shown in Table 1). Some of the LF s positive effects are immune regulation (immunosuppression or immune activation), regulating redox (i.e., oxygen free radicals releasing processes), inhibiting cancer cell growth, and LF has been shown to induce apoptosis in cancer cells. It has also been determined in some research that the LF induce apoptosis in cytotoxic T cells and NK cells. As a result of its ability to cross the blood-brain barrier, it is assumed that LF s can be used as a carrier for chemotherapeutic agents in the treatment of brain tumors. This hypothesis may not only enhance the effects of chemotherapy drugs but also reduce the frequency of unwanted side effects [10].

LF supplementation with colostrum has a major role in the development of infants and also elderly patients during convalescence after serious diseases. LF damages the cell structures of the microbes causing osmotic disruption and restricted nutritional intake. Additionally, by preventing pathogens from binding the epithelial cells LF inhibits infection development [28]. Moreover, the effects of LF against various acute respiratory infections related to SARS-COV-2, which causes COVID-19, have been examined. In this research, LFs immunomodulator and anti-inflammatory effects have been observed. The main reason for this research about the effects of LF on COVID-19 is that LF is commonly used as a food adjuvant in infant formulas, and the prevalence of COVID-19 in children aged 0–10 years is 0.9% in cases recorded in China. This observation triggered research about the effects of LF on COVID-19, but further tests and research are essential for the determination whether a decisive conclusion can be drawn [29]. Furthermore, oral supplementation of LF regulates the iron homeostasis. In addition, oral supplementation of LF does not exhibit side effects like the other iron supplements. LF is essential for the growth and the development of the intestinal mucosa and is involved in the repair of intestinal damage [30]. In summary, Lf has a variety of positive effects owing to its iron-binding feature, and further research is necessary and important to determine the most effective applications of these various properties of LF.

### 2.2. Immunoglobulin G (IgG)

**Figure 1:**
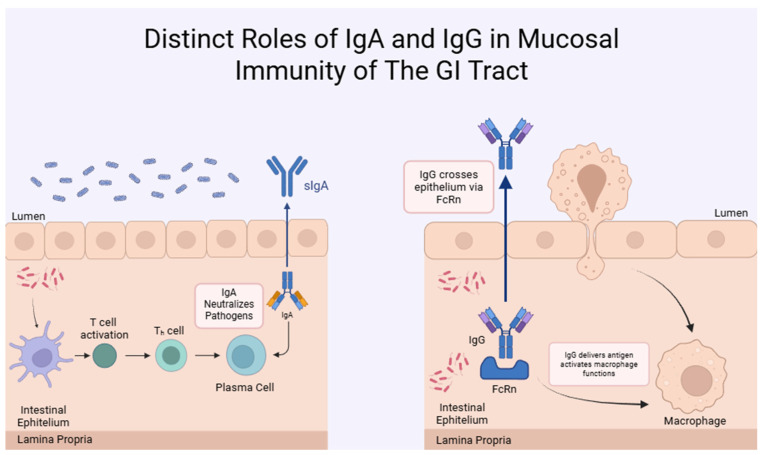
Differential Functions of IgA and IgG in GI immunity. Left Panel (IgA—Non-inflammatory barrier protection). Dendritic cells activate T cells after recognizing pathogens. The activated helper T cells (T_h_) stimulate the differentiation of plasma cells. Plasma cells produce IgA antibodies that are transported into the lumen via epithelial cells. sIgA (secretory IgA) neutralizes pathogens in the lumen, preventing them from adhering to the epithelial surface and initiating infection. This mechanism protects the mucosal surface without causing inflammation. Right Panel (IgG—Subepithelial immune regulation and inflammatory response). IgG is synthesized by plasma cells in the lamina propria and transported from the intestinal epithelium to the lumen via the FcRn receptor. In the lumen, IgG binds to pathogens and marks them (opsonization). Macrophages recognize and phagocytose IgG-labeled antigens and activate the inflammatory response. This process initiates a deeper and systemic immune response.

Among the various bioactive components of BC, another bioactive component of BC is Igs. Specifically, passive immunity is transferred to newborn calves through BC, and this transfer is mediated by Ig. In detail, transfer of passive immunity occurs through absorption of Igs through the GI tract. A study has shown that Ig absorption by epithelial cells in newborns decreases after 12 h and stops at 24 h after parturition [23]. Ig exhibits antibody activity, and their chemical structure is similar to that of antibodies. These molecules derive from plasma cells and have the functional ability to destroy pathogens. Passive immunity gained through BC provides the newborn with the necessary boost for immunity, energy, growth, and development. Moreover, extended colostrum supplementation will increase Ig concentrations, leading to enhanced immunity and elevated anti-inflammatory effects [2]. Immunologically, Igs has subclasses, including IgG, IgA, and IgM. The highest concentration among these subclasses is IgG (as shown in Table 1). IgG is essential for passive immunity transmission. Deficient BC supplementation in newborn calves leads to inadequate IgG uptake, and the newborn becomes more susceptible to diseases, which can cause major problems later. Given that passive immunity in humans is transmitted to the offspring in utero, the lower amount of Ig in human colostrum than in BC can be explained. BC’s quality is measured on many farms by the concentration of IgG in its content. This quality standard is mostly a minimum of 50 g of IgG per liter [31]. Additionally, heat treatment of BC can enhance IgG absorption and reduce bacteria. It is suggested that newborns must be administered an average of 150–200 g of IgG for adequate passive immunity. The subclasses of IgG, IgG1 and IgG2, IgG1 is the primary one, and the ratio between these two subclasses is approximately 7:1. BC also contains small amounts of IgA and IgM. The role of IgM is to recognize and neutralize pathogens in the blood. The role of IgA is to support mucosal health (as shown in Figure 1) [32]. Moreover, colostrum obtained from cows is used for the BC supplements. If colostrum samples are to be taken from cows vaccinated against pathogens, the specificity of IgG in BC may be enhanced. IgG is a glycoprotein in polymer or monomer form, and IgG also has high immune activity and is biologically active. Thus, IgG, which has a high immune effect, can directly bind to the virus, and it can neutralize the toxicity of bacterial toxins by promoting phagocytosis of macrophages against antigens, attachment of pathogens to the intestinal epithelium can be prevented by IgG (as shown in Figure 1), Numerous studies demonstrate that intense or prolonged physical training makes athletes more susceptible to upper respiratory tract infections. BC has various clinical or therapeutic applications in humans. Several studies have revealed that oral supplementation of BC can reduce the incidence of these respiratory tract conditions. According to one of the referenced studies, BC supplementation for 20 days increased s IgA (salivary IgA) concentration by 33% [33]. Thus, the increase in IgA enhances immunity and contributes to the decreased incidence [34]. Igs are essential bioactive components of BC, especially IgG is vital for the newborn calves due to its passive immunity transmission properties.

### 2.3. Growth Factors

A different bioactive component of BC is GFs. In general terms, the biological functions of GFs include promoting wound healing, facilitating muscle and bone development, and stimulating cell proliferation. The concentration of GFs present in BC differs based on their biological sources. The average concentrations of these GFs in BC are presented in Table 1. The GFs that are emphasized in this paper due to their effects on the GI tract are IGF-1 and TGF-β. Moreover, approximately 50 polypeptides present in BC as GFs. New methods are being developed to extract these GFs. Furthermore, similar to Igs, the concentration of GFs present in BC varies within hours after birth. TGF-β is a key GF involved in the regulation and repair of the GI tract. TGF-β has a variety of biological functions, these functions include oncogenesis, suppression of immune responses, and cell proliferation. In addition, these functions are mostly expressed in the GI tract. TGF-β exhibits anti-inflammatory and immunomodulatory properties and acts as a barrier to protect the intestinal epithelium. Additionally, TGF-β has significant functions in maintaining intestinal balance and plays a role in the regulation of innate immunity [35]. There is 100% sequence similarity between human colostrum and BC. The highest concentration of GFs in BC is IGF-1. IGF-1 promotes the growth and differentiation of cells and tissues. Therefore, several animal studies demonstrate that IGF-1 is a key factor in the repair of intestinal epithelium after damage. Although some studies indicate that colostrum supplementation results in an increase in IGF-1 concentration, on the other hand, different studies report that BC supplementation does not cause any effect on IGF-1 concentration. In addition, some studies have also reported that colostrum may reduce intestinal permeability by promoting enterocyte development [36].

### 2.4. Oligosaccharides

BC’s other essential bioactive components are oligosaccharides (OS) and glycans. IgG plays the predominant role in terms of immune effects in BC; however, there are also specialized bioactive components to promote the development and maturation of the newborn. BC is abundant in these components, which makes it an appropriate supplement and a therapy for GI disorders. Among the specialized bioactive components, OS stands out due to its specific functions. BC contains high concentrations of OS that prevent pathogens from adhering to the intestinal epithelium, enhance IgG absorption, and positively affect the growth of beneficial bacteria with its prebiotic functions. Compared to OSs in human milk, more than 70% of the OSs in BC contain one or more sialic acid residues. Additionally, naturally selected bovine OSs can promote the formation of healthy gut microbiota when ingested through colostrum [37]. Moreover, these OSs provide a wide range of benefits to newborns with their prebiotic properties. OS can promote the growth of beneficial Bifidobacterium and positively affect gut health. Some studies conducted on mice demonstrated that OSs increase the abundance of Bifidobacteria and microbiota balance by reducing permeability and inflammation. Therefore, Bifidobacteria make up a large proportion of the newborn microbiota, which means that BC have a key role in neonatal health, and evidence suggests a connection between early nutrition and later animal production yield; therefore, OSs are one of the essential bioactive components in BC [26]. Another bioactive component of BC that can be mentioned in terms of its prebiotic features is *N*-glycans linked to glycoproteins. In one study, *N*-linked glycans were found to be a growth substrate for *Bifidobacterium infantis* (*B. infantis*), nonetheless, current understanding is limited on whether free OSs are a better substrate for *B. infantis* than glycoconjugates. To investigate this, *N*-glycans were released with EndoBI-1 in the laboratory. Thus, in these studies, it was known that *N*-linked glycoproteins were used as growth substrates for this beneficial *bifidobacterium*; however, it was still unknown whether free OSs were better growth substrates. Briefly, in this study, it was determined that the released *N*-glycans are the glycans that provide high growth efficiency for *B. infantis.* In this study prebiotic properties of *N*-glycans contained in BC can be determined. When these glycoprotein-bound *N*-glycans remain in glycoproteins in protein-bound form in the microbiota, no prebiotic effect is observed because they are not cleaved as efficiently as EndoBI, and this leads to low yields of prebiotic activities. Therefore, endoglycosidases such as EndoBI-1 can be used to liberate these glycoprotein-bound *N*-glycans [38]. In summary, the prebiotic features of OSs and glycans present in BC, and the role of these components in maintaining microbiota balance make it an important bioactive component that requires further research and study.

### 2.5. Bioactive Peptides

Among the bioactive peptides in BC that stand out for their effects on gut health are alpha lactalbumin, caseins, beta-lactoglobulins, and osteopontins. α-Lactalbumin (α-LA) is a low molecular weight, calcium-binding, tightly structured protein. It has a key role in the BC due to its many physiological functions and rich nutritional content. Also, α-LA plays a role in cancer prevention and treatment by binding with oleic acid. Bioactive peptides obtained by digesting α-LA have many positive effects on human health. These effects can be identified as antimicrobial, antiviral, anti-inflammatory, anti-ulcer, antihypertensive, and antioxidant. Further research is required to determine the specialized mechanisms of action of α-LA in the human body [39]. Therefore, α-LA has immunomodulatory activities. Peptides produced by a-LA digestion may exhibit antimicrobial activity and affect epithelial regeneration through selective apoptosis. In addition, the peptides released during this digestion can also exert immunostimulatory effects in human and mouse macrophages. In one study, α-LA supplementation was shown to diversify the gut microbiota and promote the growth of some beneficial bacteria. To summarize, α-LA positively affects gut function, microbiota, and immunity [40].

Caseins are one of the essential peptides in BC. Caseins can be isolated from other proteins by methods such as electrophoresis, chromatography, and enzymatic processes. In addition, caseins are known as irregularly structured proteins with no clearly defined secondary structures. Caseins with amphiphilic structures naturally assemble when dispersed in aqueous solution, form calcium phosphate bridges through hydrophobic interactions, and generating stable micelle structures; this feature contributes to the maintenance of micelle integrity. According to whether the bovine from which BC is obtained is an A1 or A2 variant, the form of β casein it contains differs. This difference results in an amino acid sequence alteration in the β-casein of A1 bovines. Consequently, this alteration in the amino acid sequence leads to the release of a peptide called beta-casomorphin-7 (BCM-7) as a result of the digestion of A1 β-casein. The release of this compound is associated with several clinical disorders, including abnormal GI function, cardiovascular disease, type 1 diabetes, schizophrenia, and autism, and these properties of BCM-7 have been widely studied in medicine. However, whether BCM-7 is directly involved in these diseases has not been proven, but observations suggest that it may be. Further research should be conducted to determine the mechanisms of action of this compound at the molecular level [41]. On the other hand, the digestion of A2-derived caseins has been demonstrated to reduce BCM-7 formation and its associated complications. In a study that was conducted on mice, the effects of A2 β-casein were revealed. The results showed that, A2 b-casein digestion resulted in a change in the composition of the intestinal microbiota with an increase in bacteria such as *Ruminococcaceae*. Moreover, the immune system improved, and intestinal absorption and intestinal function were enhanced. In light of the studies, the bioactive caseins in BC have positive effects on the intestinal microbiota, enhanced intestinal function, and promoted absorption [42]. Beta-lactoglobulin stands out with antimicrobial and anticarcinogenic properties. Beta-lactoglobulins prevent bacteria from adhering to the surface of the host cell, thus exhibiting antimicrobial properties. These proteins have various beneficial nutritional and food functions. Beta-lactoglobulins are also thought to protect against the formation of tumor precursors in the intestinal wall; this demonstrates their anticancer features [43]. Beta-lactoglobulins can alter the barrier function of intestinal epithelial cells, especially through complexes formed with polyphenols. These complexes can reduce permeability by protecting the intestinal barrier [44].

## 3. Mechanisms of Action in the GI Tract

GI diseases are among the most common diseases that affect a large part of the population and for which people seek medical treatment. These diseases are often triggered by pathogen-induced infections, inadequate and unhealthy diets, stress, and medications with side effects. Different approaches to the treatment of these diseases are being considered. For instance, diet-based approaches are particularly prominent, and BC stands out among these diet-based approaches with some of its properties. In addition, the mechanisms of action and therapeutic properties of BC on the GI tract need to be elucidated and analyzed to benefit from this therapeutic property of BC. Moreover, BC has a higher therapeutic potential than mature milk due to its greater concentrations of Igs and antimicrobial factors [6].

### 3.1. Mucosal Barrier Protection

The gut represents an ecological niche with a balanced microbiota serving as a habitat for a large number of bacteria, viruses, protozoa, and fungi. The nutrients consumed contain these organisms and may also contain pathogens. Mucosal barrier refers to the anatomical and functional boundary between the host and the intestinal lumen. The tissue repair speed of the intestinal epithelium is very high, so cells damaged by pathogens are regenerated in a short time [45]. BC is a nutritional component with various properties that contribute to mucosal healing. For instance, many diseases in the GI tract are thought to be caused by impaired mucosal integrity. BC enhances mucosal barrier repair through several mechanisms [44]. Moreover, the absorptive and barrier functions of the intestinal mucosa are essential for intestinal health. Healthy development of the intestine is critical for this barrier function. This barrier provides communication between the inside and outside of the cell owing to its permeability. This function of the barrier is one of the most essential mechanisms for intestinal health, and the formation of this barrier consists of a high proportion of tight junction proteins. Additionally, damage to this permeable barrier may result in the passage of certain pathogens and detrimental compounds. This passage can lead to inflammation in the intestine, a condition known as leaky gut syndrome. Therefore, BC can be considered a functional nutrient with significant benefits in maintaining the mucosal barrier. EGFs, which are present in BC, play a role in strengthening these tight junction proteins. In other words, this function of EGFs in BC may reduce the risk of leaky gut syndrome. In mammals, unimpaired intestinal permeability is higher in newborns. In addition, a study conducted in rabbits, the effect of BC supplementation on the intestinal barrier was observed and BC supplementation was shown to promote growth over the barrier [46]. The mechanism of action of EGF on tight junction proteins has been observed in various studies. These studies demonstrate that EGF binds to the EGF receptor and activates Ras/MAPK, PI 3K/AKT, PLC-γ/PKC and STAS signaling pathways and thus regulates intestinal barrier function. Among these signalling pathways, EGFR-phospholipase (PLC)-γ-PKC and EGFR-ERK/MAPK play a role in maintaining tight junctions through EGF. Consequently, the ERK/MAPK signaling pathway increases gene expression of tight junction proteins such as ZO-1, claudin-1, and occludin [47]. As a result of one of the studies, it can be concluded that the increased function of tight junction proteins via EGF is dependent on ERK1/2 activity [48]. Furthermore, TGF-β signaling acts as an important regulator for the maintenance of epithelial barrier function in intestinal barrier cells. This regulatory function is mediated by the modulation of gene expression of tight junction proteins. The signaling pathway of TGF-β is responsible for the regulation of transcription of some *claudin* gene family proteins, such as *Cldn2*, *Cldn4*, *Cldn7*, and *Cldn8*, which have key roles in maintaining permeability and cellular balance. Thus, this regulation by TGF-β is dependent on RNA transcription for *Cldn2*, *Cldn4*, and *Cldn8* genes. This leads to the effect of TGF-β being presented at the transcriptional stage and not in the increase of new mRNA synthesis mechanisms. According to this information, it can be concluded that TGF-β signaling has multifaceted effects on the intestinal epithelium and can regulate and alter the tight junctional structures at the genetic level [49].

**Figure 2:**
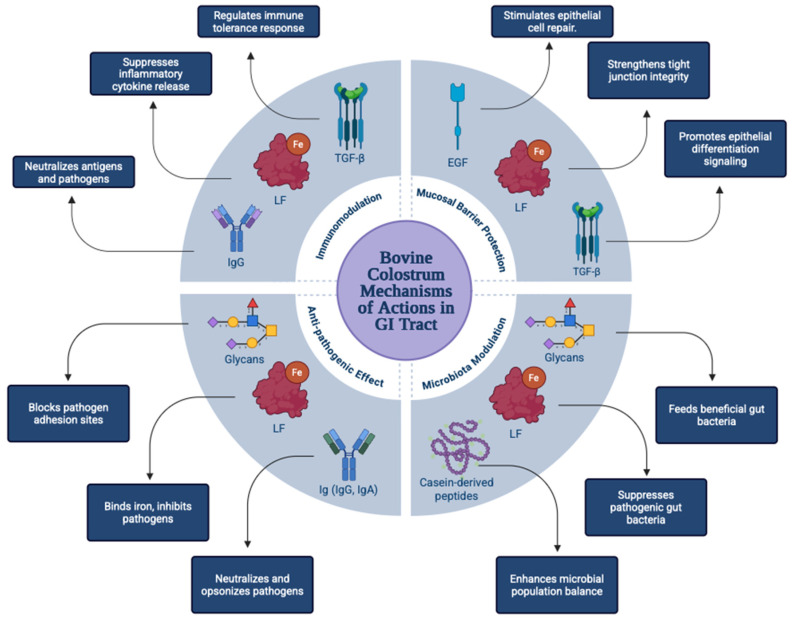
Bovine Colostrum Mechanisms of Action in the GI Tract. This figure visually summarizes the four main mechanisms of action of BC in the GI tract (mucosal barrier protection, immune modulation, antipathogenic effects, and microbiota regulation) and the major bioactive components involved in these processes.

BC is a compound rich in glycoproteins called LF. LF stands out with its protective properties against intestinal barrier disorders that develop under the influence of aflatoxin M1 (AFM1). This property of LF has been observed in both in vivo and in vitro studies, and these findings consistently support its barrier-protective feature. In these studies, it was observed that LF is highly effective in the recovery and preservation of intestinal barrier integrity, and this mechanism of action was determined to be realized by regulating the expression of tight junction proteins in the intestinal epithelium and restoring the structure of the epithelium. In addition, in case of disruption of tight junction structures caused by AFM1, LF supplementation corrects these tight junction structure disorders by re-expressing and correctly localizing the components that are essential in the barrier, such as claudin-3, occludin, and ZO-1 (as shown in Figure 2) [50]. Moreover, based on these findings, the function and mechanisms of action of LF on intestinal epithelial damage and maintenance of the intestinal barrier can be understood more clearly. For instance, in a study conducted on mice, the effect of LF on intestinal epithelial damage and restoration of intestinal barrier function in mice exposed to toxins such as deoxynivalenol (DON) were demonstrated. This effect is mainly due to the reorganization of tight junction proteins and inhibition of the MAPK signaling pathway. In the intestines of mice exposed to DON, the expression of tight junction proteins such as occludin is disrupted, and intestinal permeability increases. To improve these complications, the expression of tight junction proteins such as occludin must be increased. LFs play a major role in increasing this expression, and by increasing the levels of these proteins, intestinal barrier integrity is restored. This restorative effect is mediated by the suppression of p38 and ERK1/2 phosphorylation by LFs. Furthermore, this has been demonstrated in mice in which barrier disruption of the inflammatory response caused by activation of the MAPK signaling pathway is reversed by LF [51]. Briefly, LF maintain barrier integrity by increasing the synthesis of tight junction proteins and cell localization of these proteins, suppressing inflammation, maintaining mucosal integrity, and stabilizing epithelial cell survival.

### 3.2. Immunomodulation

BC has bioactive components with various positive effects on immunity. BC contains proteins with antimicrobial properties, such as IgGs and IgAs, GFs, LFs, and other proteins with immune- supportive effects. To contribute to passive immunity with these components, these nutrients must be able to function in the system of the individuals receiving BC supplements in a certain harmony and in a suitable environment. Moreover, these components maintain the balance of intestinal microbiota, have anti-inflammatory effects, and show immunomodulatory functions with these properties. Studies have shown that Ig, cytokines, and GFs in BC have mechanisms of action that regulate immune responses at the mucosal level. In the remainder of this subsection, the mechanisms of action of the major immunomodulatory components of BC are discussed [5].

- Immunoglobulins (Igs)

One of the most highly concentrated immune factors in BC is Ig. Among the Ig classes, it is the IgG class (especially the IgG1 subclass) that exerts the greatest immune effect, but IgM and IgA also have several important effects. IgGs are the most essential components for passive immunity transmission in newborn cattle because cows do not pass IgG through the placenta, and newborn calves have no innate Ig [2]. This passive immune transmission provides pathogen neutralization and can prevent the local cytokine secretion caused by infections at an early stage. BC quality is usually determined by IgG concentration, but some studies have demonstrated that calves fed colostrum containing all components show more enhanced immune responses than those fed serum containing only IgG. Based on the results of these studies, it is thought that Ig goes beyond binding antigens and plays a role in the regulation of various cytokine responses by interacting with other components in BC. For example, IgG/IgA inhibits the stimulation of pro-inflammatory cytokines such as TNF-α and IL-6 in the intestine by reducing pathogen load and toxins [5]. In addition, in a study conducted on colostrum samples obtained by milking immediately after birth, concentration changes of bioactive components in BC content and biological activity changes depending on this concentration change were observed. In the light of this study, it can be determined that IgG and cytokine concentrations such as IL-1β, IL-6, TNF-α decrease until the third day. Based on this information, it can be inferred that the effects of Ig in colostrum, especially IgG, on immunomodulation are most intense during milking immediately after birth [19]. Additionally, after oral BC supplementation, particularly IgG can reach the intestine and provide local immunomodulatory effects. Oral supplementation of bovine IgG purified from the colostrum of cows immunized with recombinant TNF-α, and the fact that this antibody remains active in the GI tract and provides local effects, can be given as an example of the effects of IgG in BC on immunomodulation [52].

- Growth Factors

IGF is an important bioactive component in the repair of the epithelial barrier in BC. Locally, IGF-1 activates P13K7AKT and MAPK signalling pathways in enterocytes, thereby suppressing epithelial proliferation and apoptosis [53]. Due to these effects, IL-6 and TNF-α production is reduced, and a barrier function is created to support the prevention of inflammation. Systemically, IGF-1 alters the function of macrophages and balances the production of IL-2 and IFN-γ, providing supportive effects on immune responses. In an experimental supplementation in preterm pigs, primarily those with the lowest levels of IGF-1, these levels were also associated with various immune markers. IGF-1 supplementation suppressed plasma IL-10, IFN-γ, and IL-2 responses in these premature piglets and reduced the expression of genes related to Th1 polarization. As shown in a study, IGF-1 exerts immunomodulatory effects on the systemic immune system through various mechanisms of action, such as suppression of Th1 expression, reduction of proinflammatory cytokines, and increase in anti-inflammatory IL-10 [54].

Another bioactive molecule in BC that has immunomodulatory functions is TGF-β. TGF-β signaling has a critical effect on the regulation of immune responses by inducing tolerogenic properties in dendritic cells (DC). Studies have shown that TGF-β signaling reduces tolerogenic properties in DC. DCs lacking TGF-β receptor-II exhibit proinflammatory functions, active lymphocytes, and limit and inhibit the differentiation of Treg cells, the regulatory T cells that are specific for antigens. Furthermore, this leads to multiple-bit autoimmunity in organs. Based on the studies, it can be determined that a strong TGF-β signaling pathway is essential in the immunity of the organisms, especially in immunomodulation. In addition, in a study, it was determined that the effect of the synthetic TGF-β agonist T74 on the DCs mentioned through the TGFBRI pathway, that is, it causes inhibition of cell maturation and increases cellular tolerance [55].

- LF

LF has immunomodulatory functions that offer positive effects on both innate and acquired immunity. LF binds to and neutralizes endotoxins such as lipopolysaccharides (LPS) and thus plays a regulatory role in immune responses. This mechanism of LF also prevents LPS from increasing in the intestinal tissue and bloodstream. In addition, LF decreases gene expression of proinflammatory cytokines such as TNF-α, IL-1, and IL-6 and suppresses the effects of inflammatory cells. Moreover, to this function, LF shows immunomodulatory effects by playing a role in increasing the release of anti-inflammatory cytokines such as IL-10 and defense system-stimulating cytokines such as IL-12. In addition to these effects, LF effects on immunity are supported by functions such as suppression of NF-κB and MAPK signaling pathways, reduction of oxidative stress, and protection of the integrity of the cellular barrier. LF enhances various effects of granulocytes, lymphocytes, macrophages, and NK cells and increases the functional capacity of immune cells [56].

### 3.3. Anti-Pathogenic Effects

BC contains several bioactive components with high levels of anti-pathogenic activity and immune factors such as LF, IgG, IGF, and EGF. Moreover, BC products may inhibit the growth of pathogens in vitro and stimulate epithelial cell proliferation to maintain intestinal epithelial integrity. In a study, BC with gentle thermal processing used as a fortifier in HDM (donated human milk) demonstrated beneficial effects on the level of bioactive proteins and antimicrobial activity against pathogens such as Staphylococcus epidermidis, *Escherichia coli*, and *Enterococcus faecalis*, which are pathogens causing neonatal sepsis. In this study, these effects were observed by comparing unfortified human milk with HDM supplemented with BC as a fortifier. As a result of this study, the addition of BC fortifier to human milk prevented in vitro overgrowth of bacteria involved in neonatal sepsis [57].

LFs are iron-binding proteins of the transferrin family. It is synthesized from the epithelium of various organs in the body. Due to this feature of BC, LFs are found in the content of external secretions such as antibacterial, antiviral, antifungal, and antiparasitic, as well as antitumoral, anti-inflammatory, immunomodulatory, and antioxidant activities [8]. If the scope of the studies is evaluated, it can be determined that the antibacterial properties of LFs are the most extensively studied property. LFs and LF-derived peptides show a wide range of biological activity against Gram-positive and Gram-negative bacteria, but the range of activity of LFs is not limited to the same LFs, and LF-derived peptides are also effective against viruses, fungi, yeast, and parasites. A large part of the mechanisms of action of these antimicrobial activities of LF is related to the iron-binding property of LF. When the LF structure is examined, it contains four domains, N-1 and N-2, C-1 and C-2; each of these domain pairs carries a deep iron-binding pocket. By studying LFs at the molecular level, it can be explained how they acquire this iron-binding function. In addition, LF-derived peptides such as lactoferricin and shorter lactoferricins also have antibacterial and antiviral properties [58]. In a study, BC-derived LFs had an antibacterial effect on *Pseudomonas aeruginosa* depending on the dose range of LF that was used. In the light of this study, *S. aureus* bacteria showed resistance to the antibacterial effects of LF regardless of the dose range. Based on this study, it can be said that the antibacterial activity of different doses of LF may be different, and this antibacterial activity is not valid for all bacteria [59]. LF has a high affinity for iron; this affinity for iron facilitates the maintenance of iron homeostasis in the body, thus limiting the formation of free radicals and preventing dysfunctions in the chelation process that reduce this excess in case of iron in organs. Furthermore, this property of LF limits iron access for pathogens that need iron for growth, which is one of the mechanisms of action against pathogens [60]. LFs are usually not completely saturated with iron but can reach higher iron concentrations due to different diets and overall iron levels altered by diseases. These iron-saturated LFs can be called holo-LFs, and this form can exert microbicidal effects to protect the organisms from certain pathogens. Moreover, the iron-free apo-LF form can also exert a microbiostatic effect, i.e., inhibit the growth of bacteria, through its iron-binding function, an effect that may explain the antimicrobial and antibacterial mechanism of the iron-binding property of LF. LF binds to apo-LF lipopolysaccharides, porins, and other proteins in the outer membrane of many gram-negative bacteria. As a result of this binding, the permeability of the membranes of pathogens increases, and this mechanism leads to lysis and cell death of pathogens. Some molecules derived from LF, like lactoferricins, also have the ability to bind to proteins in the outer membrane; this binding and subsequent release effect make LF an effective bioactive component for pathogen protection [61]. Additionally, LF also have antiviral properties; in vitro studies on SARS-CoV-2 have demonstrated that Bovine LF inhibit the early phase of infection [62]. As mentioned in previous mechanisms, LF also suppress viral replication processes by restricting access to iron binding, and oxidative stress is reduced, thus minimizing the damage to the cell. With its cationic structure, Bovine LFs have a high affinity for components with anionic surface structure. These electrostatic interactions are one of the mechanisms that explain the adhesion of LF to surfaces. Therefore, LF act as a barrier on the surfaces to which they adhere, and with this function, LF have effective mechanisms to prevent the onset of viral infections by reducing the adhesion of viral agents to the host and their passage into the cell [63]. In addition, by decreasing IL-6 levels caused by viral infections and inflammations resulting from these infections, LF contributes to both keeping the activation level of the immune responses in balance and maintaining iron balance [64].

Ig is another bioactive component of BC that stands out for its antimicrobial effects. The effects of Ig on the immune system and its mechanisms against infections have been under investigation for a long time, and various findings on the therapeutic and protective properties of Ig have been obtained. Ig has defense mechanisms not only against pathogens but also against various allergens. The main function of Ig against pathogens on mucosal surfaces is to bind specifically to these pathogens and prevent their passage through the epithelial barrier, thus limiting the onset of infections at the initial stage. Besides, Igs prevent pathogens from adhering to the intestinal epithelium, and this mechanism is called immune exclusion [21]. In some studies, in contrast to IgA, which is thought to prevent the passage of antigens through the mucosal barrier, it has been suggested that IgG forms complex structures that are more easily taken up by epithelial cells and offers a positive effect to the immune system [65]. During bacterial infection, the effects of Ig against pathogens are very important because when Ig binds to antigens on the surface, in addition to direct recognition of these antigens, Ig also activates the complement system and promotes the degradation of pathogens by initiating phagocytosis by neutrophils and macrophages. This mechanism reduces the spread of infections by providing a positive effect on the neutralization of bacterial toxins [66]. IgA stands out with its important antipathogenic function. The antipathogenic mechanisms of action of IgA are not only to determine the bacterial species present in the gut microbiota, but also to regulate the localization of these bacteria in the microbiota [67]. This function causes IgA to keep the physical contact between the microbiota and the immune system at the lowest level, thus minimizing the negative effects that may occur in case of contact. In an IgA-deficient organism, this function is lost, and thus the composition of the gut microbiota is disrupted, and translocations of microbial components are increased. This leads to increased inflammatory responses, and the ability to control the interaction between pathogens and mucosa is weakened; this disrupts the microbial balance [68].

Furthermore, glycans in BC have selective prebiotic activities, and this selective activity indicates that glycans play a major role in regulating microbiota balance. Due to these functions, among the anti-pathogenic effects of BC, OSs prevent the binding of pathogens to the host cell with their composition-regulating effects [69]. Owing to the glycans in the BC content, colonization of beneficial bacteria is promoted, and thus, competitive interactions between beneficial bacteria and pathogens occur. This leads to the indirect anti-pathogenic effect mechanism of OS [38]. In addition, this effect is symbiotic, as glycans and OSs act as competitive inhibitors of pathogen binding to mucosal surfaces in the GI tract [69]. Together with its bioactive components, BC provides the organism with protection against pathogens, which makes it easier for the immune system to mature healthily and maintains the balance of the microbiota [70]. The BC components that are more prominent with these effects are LF and Ig, but also OS and GFs have functions on anti-pathogenic effects [70].

### 3.4. Microbiota Modulation

BC consumption is critical for the healthy development of the microbiota composition in newborn calves, and subsequent BC supplementation has positive effects on this microbiota composition. In particular, BC has various effects on bacterial colonization in the gut microbiota. In one study, colonization of enterotoxigenic *Escherichia coli* in the intestine decreased with specific OS supplementation in newborn pigs [71]. BC shows this colonization effect not only on pathogens but also on various *bifidobacteria* and *lactobacilli* beneficial to the gut microbiota. In addition, some studies have demonstrated that BC alters the intestinal cell surface and thus affects the attachment of *Bifidobacterium* and *Lactobacillus* species [72]. BC exerts these effects with the bioactive components in its content, and these bioactive components are the components that help to support the intestinal microbiota with beneficial flora, with various prebiotic effects. In terms of this effect, OSs and OS-derived glycans stand out [25]. OSs are present in large amounts in BC. The more predominant of these OS in BC are 6′-sialyllactosamine, 6′-sialyllactose, 3′-sialyllactose, and disialyllactose. Furthermore, there are glycan structures derived from BC that contain high amounts of *N*-glycolyzed proteins. These *N*-glycan structures are seen as resources that can play a very important role for prebiotic substrates and may become more important with further studies [73]. BC exhibits prebiotic activity through *N*-glycans that show selective utilization by specific bacterial species in the GI microbiota of adults. Using this selectivity, the composition of the microbiota can be regulated in a targeted manner, and a significant advantage can be achieved [11]. Moreover, the mechanism of prebiotic activity of OSs can be explained by the specific promotion of the growth of beneficial *Bifidobacterium* strains such as *B. infantis*, *B. breve*, and *B. bifidum* and various *Lactobacillus* species [74]. In an in vitro study, it has been shown that the structural differences among OSs determine the amount and type of OSs used by beneficial bacteria in the gut microbiome. This suggests that OSs have selective and targeted effects [75]. The selective bacterial growth stimulation of OSs mentioned above is characterized by its effects on the proliferation of beneficial bacteria. Specific Bifidobacterium species have been shown to reduce oxidative stress and improve gut morphology. Additionally, OSs have been shown to increase the relative abundance of predominantly beneficial bacterial species in both the small and large intestinal lumen [76]. With these effects of OSs, it was determined in a study conducted on piglets that OS supplementation reduced diarrhea symptoms and promoted growth [77]. In a different study, it was observed that bovine-derived OS supplementation in neonatal pigs led to both improved growth and changes in fecal microbiota to enhance nutrient utilization [76].

## 4. Evidence from Preclinical and Clinical Studies

### 4.1. Infectious Diarrhea

As mentioned in previous sections, BC has a very rich content of bioactive components and a unique composition of nutrients. Due to these properties, BC has long been used in many species not only as an additional nutritional supplement but also as a supportive element with preventive potential in the treatment of various infectious diseases such as diarrhea. These infectious diseases are caused by specific pathogens, including viral agents such as rotavirus and coronaviruses, protozoa such as *Cryptosporidium parvum*, and bacterial pathogens such as enterotoxigenic *Escherichia coli*, *Clostridium perfringens*, and *Salmonella* species. The bioactive compounds in BC may have protective effects against these pathogens [78]. In this section, the effects of BC supplementation on infectious diarrhea are supported by clinical studies.

Diarrhea is one of the major factors of high morbidity and mortality rates in dairy calves. Most of the diarrhea cases in calves are treated with antimicrobials, but this treatment method increases the risk of antimicrobial resistance over time and brings up the need to turn to more sustainable alternative treatment methods in the prevention of diarrhea cases. In a study, the median recovery time of diarrhea cases was 3.5 days in the control (CON) group, 2.75 days in the standard treatment control (STC) group, and 2.75 days in the colostrum treated (LTC) group (Table 2). As a result of these analyses, it was determined that the diarrhea effects of calves in the LTC group decreased faster compared to the CON group. In addition, body weight gain was determined by linear regression method, and it was determined that calves in the LTC group grew 98 g more per day compared to the CON group for a certain period of time [79]. The findings of this study indicate that BC supplementation may be an alternative treatment for diarrhea.

In another systematic review, five randomized controlled trials were included in the meta-analysis and evaluated the effects of BC and its derived compounds against infectious diarrhea in a total of 324 children. Compared to the placebo group, BC supplementation decreased the frequency of defecation, decreased the incidence of diarrhea after supplementation, and decreased the detection of rotavirus and *E. coli* in feces. The outcome reports of the trials showed a reduction in the incidence of respiratory tract infections, diarrhea and hospitalization in children receiving four weeks of BC treatment. Randomized controlled trials have shown benefits of hyperimmune BC in rotavirus-induced infectious diarrhea that should not be ignored. Moreover, hyperimmune BC has been shown to be more effective against infectious diarrhea than regular BC, but this effect does not apply to *Shigella* -associated diarrhea. Of the studies included in this meta-analysis, one included BC, two included hyperimmune BC and two included hyperimmune BC- derived Ig administration. Hyperimmune BC resulted in a significant reduction in the incidence of diarrhea (OR = 0.32, 95% Cl: = 15–0.67) and was associated with a parallel reduction in stool frequency, diarrhea incidence and pathogen positivity. Furthermore, from this analysis, BC supplementation has an effect on the frequency of defecation, which decreases to an average of once a day [80].

In formula-fed infants, a decrease in the frequency of watery stools, frequent stools, loss of appetite, sneezing, upper respiratory tract infections and diarrhea was observed after three months of BC supplementation. In addition, the duration of diarrhea was shorter. No adverse reactions associated with BC were reported, suggesting that it is suitable even for children with milk intolerance. BC reduced diarrhea symptoms, shortened the duration of illness, and accelerated recovery. A significant reduction in GI infections was observed after three months of BC supplementation and BC containing 200 mg Ig daily was reported to be effective in preventing diarrhea. In rotavirus-induced diarrhea, infants receiving BC supplements gained 403 g of weight in four days, compared with 343 g in the control group (Table 2). However, some studies have reported no effect of BC on weight gain; therefore, further research is needed [82].

A study was conducted to identify cases of *S. dysenteriae* type 1, including children with long-standing illness. Children enrolled before the study were not given HBC or BC. Participants received 100 mL of BC three times a day for three days (Table 2). Duration of diarrhea was measured as 90 h in the HBC group and 99 h in the BC group. On the third day, the number of stools were 13 in HBC and 17 in BC; on the fifth day, it was 78 and 98, respectively. By the fifth day, positive stool cultures were less frequent in the HBC group. Children receiving HBC showed noticeable improvements, including reduced anorexia, tenesmus, diarrhea, and overall stool frequency. A marked decrease in overall symptoms was observed. However, due to the limited number of participants in each group, these favorable trends did not reach statistical significance, and no definitive antimicrobial benefit of HBC could be established [83].

In a study examining the combination of BC and egg for infectious diarrhea, BC was collected within the first 24 h after birth, because after this time, there was a rapid decrease in bioactive components such as IgG. The study tested common bacterial strains associated with SIBO, as well as EPEC and *Salmonella*, which have toxic properties (Table 2). The purpose of including these two pathogens was to determine whether there were common protective mechanisms in pathogens with different toxicity. It was observed that the combination of BC and egg did not provide a synergistic benefit, which may be due to the absence of immune cells in the in vitro model. However, BC alone or in combination with egg contributed to the maintenance of mucosal barrier integrity; bacterial translocation and apoptosis decreased, Hsp70 levels and cell adhesion molecules increased. The study demonstrated the potential benefits of BC and egg combination against infectious diarrhea and NSAID-induced small intestinal damage [84].

The oral use of BC and serum-derived Igs in combination with human serum samples has been supported by clinical evaluation as a safe and effective method against microbial infections. HBC has been found particularly useful in infectious diarrhea in children. Studies with models such as enterotoxigenic *Escherichia coli* (ETEC) have shown the potential of HBC to prevent or treat intestinal infections. Positive effects of HBC on the immune system have also been investigated. Protective effects against pathogens such as EPEC, *Salmonella*, and *Shigella flexneri* have been reported. In rotavirus mouse models, the combination of HBC with *Lactobacillus* was found to be more effective than HBC alone. Prophylactic use of Travelan^®^ based on HBC prevented the development of *shigellosis* in 75% of cases and showed high efficacy against ETEC (Table 2). It also showed cross-reactivity against G ram-negative enteric pathogens. Travelan^®^ was effective against four *Shigella* toxins and antigens in both in vivo and in vitro models [86].

In another study, infections caused by bovine rotavirus—one of the primary causes of diarrhea in neonatal calves—were investigated. The study, conducted on calves from various dairy farms in Addis Ababa, revealed a notable incidence of diarrhea. *Bovine rotavirus* was detected among the affected animals, and a statistically significant association was found between rotavirus-induced diarrhea, the timing of BC administration, and the sex of the calves (*p* < 0.05). Among all cases with rotavirus, herds with deaths were found in small-scale farms. Similar to this study, in another study, *Bovine rotavirus* was detected in 9.5% of calves with diarrhea (Table 2). Based on this study, BC supplementation may offer positive effects in the treatment of rotavirus-induced diarrhea or as a supportive supplement. However, further studies on other infectious and non-infectious causes of neonatal diarrhea and the effects of BC supplementation in the prevention of these diarrhea cases are needed [87].

Cryptosporidiosis is one of the major infections causing diarrhea and is associated with severe morbidity and mortality in calves. *Cryptosporidium parvum* infections can occur in calves shortly after birth, and calves are at particularly high risk of infection during the first 30 days of life. Bovine cryptosporidiosis causes economic losses in infected farms due to the treatment and management of enteritis. The findings of a study emphasize that IgG levels of BC supplementation given to calves on the first day of life, play an important role in the prevention of *Cryptosporidium parvum* infections that cause diarrhea. There was a significant negative correlation between anti-*C. Parvum* IgG levels in BC and clinical cryptosporidiosis symptoms (Table 2). It was determined that the incidence of *C. parvum* infections with diarrhea was higher in the offspring of cattle with low antibody levels in BC. Moreover, it was observed that the incidence of diarrhea was greatly reduced in the offspring of cattle with higher levels of anti-*C. parvum* IgG in BC. Based on these data, passive immunity transmitted through BC is a key factor in the prevention of *Cryptosporidium*-induced diarrhea in newborn calves, and BC supplementation is critical in this regard [88].

### 4.2. Inflammatory Bowel Disease (IBD)

IBD is a chronic and recurrent disease. The etiology of this disease is still not fully elucidated. Current treatment approaches for this disease, therefore, have a limited impact, and the limited impact still points to major clinical gaps for IBD patients. Current treatment strategies are often associated with alterations in the gut microbiota and target immune system disorders that sustain chronic inflammation. Drugs used in treatments targeting these disorders include aminosalicylates, corticosteroids, immunosuppressive agents, antibiotics, and biologics such as infliximab and vedolizumab. These drugs are generally used as symptom suppressants and do not offer a permanent solution to the root cause of the disease. At this point, BC stands out as a potential alternative to these drugs. BC not only supports immunity with its bioactive components but also has components with inflammatory regulatory mechanisms of action. Based on this information, it has been suggested that BC may stabilize the increased levels of inflammatory cytokine responses. However, it is very difficult to observe BC effects on IBD. This is because the cytokines that cause IBD have a very complex structure [114]. IBD has two different subcategories, Crohn’s disease and ulcerative colitis (UC). It affects the colon, impairs the function of the mucosal barrier, and is a chronic disease. Although the mechanism of this disease is not fully understood, it is hypothesized to be related to the inflammatory response of the immune system and oxidative chain reactions [89].

The effects of BC, a natural compound against UC-induced effects, were observed in a preclinical study on mice in acetic acid-induced colitis model. In this study, which focused on the anti-inflammatory effects of BC against UC and other types of IBD, it was suggested that BC may reduce the expression of proinflammatory mediators such as IL-8, ICAM, and TNF-α. Additionally, it is stated that BC shows these effects by suppressing the NF-κB signaling pathway, which plays a role in inflammatory gene activation. It has been determined that LFs in BC play a role in epithelial repair. Oral and local administration of BC in mice with acetic acid-induced colitis resulted in a decrease in parameters such as ulcer, inflammation, tissue destruction, and crypt loss. The effects of the treatment were observed with more satisfactory results with local administration. At the clinical level, BC enemas also resulted in a decrease in histological scores and clinical symptoms. Besides these effects, BC has been suggested to have positive effects on maintaining microbiota balance and decreasing oxidative stress, which is thought to be one of the main causes of UC, by increasing superoxide dismutase (SOD) activity (as shown in Table 2). The findings of this study suggest that BC supplements have a high therapeutic potential on IBD-related inflammation and intestinal damage [89].

A double-blind, randomized trial evaluated the effect of colostrum enema in combination with mesalazine in patients with active left-sided ulcerative colitis. A reduction in symptoms and regression of disease were observed in the colostrum group, but not in the placebo group. Participants were hospitalized individuals with an episode of colitis requiring a change in treatment, and the disease was limited to the left side for enema administration. All patients received mesalazine, with the dose increased to 1.6 g in those who had previously used it. Rapid and significant clinical improvement was achieved in the colostrum group. Bioactive components in colostrum, such as EGF, TGF-α, TGF-β, and IL-1β, are thought to support epithelial healing and proliferation. There was no disease recurrence during the follow-up period. The study demonstrated the therapeutic potential of colostrum, and comparative animal and in vitro studies with steroids are recommended [90].

Trinitrobenzene sulfonic acid (TNBS) induction is an experimental IBD model that specifically mimics Crohn’s disease. In another study, the possible protective effects of short-term BC supplementation in the TNBS-induced colitis model were investigated, and the findings were evaluated. In the study, mice were supplemented with BC or saline for seven days, and then colitis induction with TNBS was performed in all groups. In this model, barrier disruption, an increase in proinflammatory cytokines, and microbiota changes are observed. As a result of these supplements, clinical symptoms, histological findings, Toll-like receptor (TLR4) expression as well as pro-inflammatory cytokine levels and intestinal microbiota balance were analyzed. BC administration was not associated with any adverse histological changes or worsening of clinical symptoms. Moreover, animals receiving BC supplementation experienced less body weight loss following TNBS exposure, indicating a potential alleviating effect on colitis-related symptoms. At the molecular level, a downregulation of key inflammatory markers and immune-related genes was observed in the BC-treated group, suggesting that BC may help modulate inflammatory responses. Overall, the findings imply that BC pretreatment could offer protective benefits in TNBS-induced colitis by influencing both gene expression and microbial dynamics associated with the disease [91].

Since IBD has no definitive treatment and is a chronic inflammatory disease, in a study BC has been proposed as an alternative approach. The immune-supportive bioactive components naturally present in BC provided the rationale for this investigation. In a TNBS-induced colitis model, mice were administered BC or saline daily for a period prior to TNBS exposure. Compared to controls, the BC-treated group exhibited reduced weight loss, improved histological outcomes, and lower expression levels of key inflammatory mediators such as TLR4, IL-1β, IL-8, and IL-10, while TNF-α levels remained unchanged. Additionally, while the control group showed notable disruptions in gut microbiota—marked by an increase in *E. coli* and *enterococci* and a decline in beneficial anaerobes like *Lactobacillus* and *Bifidobacterium* these imbalances were not observed in the BC group. These findings suggest that BC supplementation may suppress inflammation and help preserve microbial homeostasis, highlighting its potential as an anti-inflammatory agent in IBD [92].

Based on the idea that early weaning may be effective in the development of inflammatory bowel diseases, a study investigated whether BC has protective effects against this intestinal inflammation in an experimental colitis model. BC supplementation was found to reduce both clinical and histologic severity of DSS-induced colorectal inflammation. Weight loss and colon shortening were less severe in mice pre-supplemented with BV-20 compared to control groups treated with BSA or water (as shown in Table 2). The findings from this study supported that BC can be administered prophylactically, causing a reduction in symptoms in a DSS-induced colitis model. Similarly, this study confirmed the therapeutic effects of BC and BC preparations in the GI tract. Prophylactic supplementation with BV-20 reduced the activity of DSS-induced colitis. The use of BSA as an agent in the control group allowed a specific comparison of the clinical benefits of BC supplementation. In addition to this information, it has been determined that Ig-enriched BC alone is not sufficient. Based on these findings, it is thought that the bioactive components in its content may be necessary to work together for an effective therapeutic effect as a result of BC supplementation. Within the scope of the study, changes in the distribution of γδ T cells were observed after DSS exposure. These altered cells are immune functioning cells that have effects on infection and autoimmunity. An increase in the γδ T cell population in lymphoid organs was observed, and BV-20 pretreatment prevented this increase. In addition, increased levels of myeloid-derived suppressor cells (MDSCs) were detected after DSS administration. These findings suggest that BC may regulate inflammation by triggering immunomodulatory mechanisms that affect the innate immune system and may constitute a safe and effective treatment strategy. Future studies should aim to clarify the underlying mechanisms that may provide long-term protection in IBD patients [13].

In another study investigating intestinal epithelial damage associated with IBD, a colostrum-derived preparation rich in anti-*E. coli*-LPS IgG, known as IMM-124E, was used to evaluate its protective effects against bacterial translocation and inflammation. IMM-124E demonstrated significant anti-inflammatory effects in both chemically and immunologically induced colitis models. It reduced immune cell infiltration, preserved mucosal integrity, and modulated T cell populations by increasing regulatory T-cell and decreasing effector T-cell. Additionally, IMM-124E lowered systemic endotoxin levels, indicating its potential to maintain intestinal barrier function. These findings support the therapeutic use of BC-derived preparations in managing intestinal inflammation and provide insight into optimal administration strategies [93].

The anti-inflammatory effects and optimal dose range of BC were evaluated in a DSS-induced colitis model. BC administration led to a marked reduction in colitis severity, improvement in disease activity scores, and enhancement of mucosal healing. These effects were supported by decreased MPO activity and restoration of barrier function through the upregulation of tight junction proteins such as claudin-1, occludin, and ZO-1 at higher doses. BC also appeared to modulate cytokine expression, help correct immune imbalances, and regulate TLR4 signaling, potentially preventing excessive innate immune activation. Microbiota analysis indicated increased microbial diversity, with a beneficial shift in gut composition characterized by higher *Akkermansia* and lower *E. coli* levels. Altogether, these findings highlight the dose-dependent and multi-targeted therapeutic potential of BC in managing ulcerative colitis and underscore the importance of defining optimal dosing in future clinical studies [94].

Another study has shown that BC supplementation supports human GI health, but the biological mechanisms behind these effects remain unclear. In this study, an in vitro model of intestinal cell inflammation was used. In this model, the anti-inflammatory and antibacterial effects of BC were investigated. When *Caco-2* and *HT29* cells were stimulated with TNF-α, BC supplementation decreased IL-8 levels. In addition, a decrease in IL-8 expression was also observed in cells co-cultured with adherent-invasive *E. coli* depending on the concentration of supplemented colostrum. In the light of these results, it is thought that the mechanisms underlying the suppression of inflammatory response by BC may be mechanisms that suppress bacterial adhesion to cells. These findings suggest that BC may be used as a potential agent in diseases such as IBD by interrupting host-pathogen interactions and suppressing inflammatory signaling in intestinal epithelial cells [95].

### 4.3. Irritable Bowel Syndrome (IBS)

A study was designed based on the increasing recognition of the potential of nutritional approaches in combination with pharmacological approaches in the management of patients with diarrhea-predominant irritable bowel syndrome (IBS-D). In this context, the effects of serum-derived bovine Ig/protein isolate (SBI), which can also be isolated from BC, on reducing GI symptoms and improving quality of life were evaluated. Accordingly, a randomized, double-blind placebo-controlled study was conducted. Patients previously diagnosed with IBS-D according to ROMA II criteria were divided into groups of 5 g SBI, 10 g SBI or placebo for six weeks. In the group receiving 10 g SBI per day (*n* = 15), significant reductions in abdominal pain (*p* < 0.01), diarrhea frequency (*p* < 0.01), bloating (*p* < 0.05), flatulence (*p* < 0.01), urgency to defecate (*p* < 0.05) and total symptom parameters were found after treatment. Significant improvements in general symptoms were observed in patients receiving 5 g SBI per day. It should be noted that the low level of inflammation observed in IBS patients may lead to histological changes, weakening of the epithelial barrier and increased intestinal permeability. The therapeutic effect of SBI supplementation against these symptoms is suggested to occur through its mechanisms of suppressing inflammatory processes and supporting the integrity of the intestinal barrier. In this study, it was stated that the transmission of microbial or food-induced antigens to the tissue may be restricted due to decreased intestinal permeability and as a result, inflammatory responses may be suppressed (Table 2). Based on these findings, it is thought that SBI contributes to the alleviation of enteropathic symptoms by providing necessary Ig and essential nutrients. Accordingly, it is suggested that BC has potential therapeutic effects in alleviating symptoms associated with IBS-D by similar mechanisms. The bioactive components of BC have the potential to help reduce these symptoms. Existing studies show that IBS patients have marked differences in fecal microbiota compared to healthy individuals. These changes in microbial balance have negative effects on intestinal barrier function and inflammation levels. It is shown in this study that supplementation of BC and BC-derived products can reduce these effects [96].

IBS affects up to 16.8% of the Korean population; yet no standardized treatment has been established. In a double-blind, placebo-controlled study investigated whether daily intake of BC (MuKoBa™, 15 mL) could improve IBS symptoms and reduce endotoxin levels. Eighteen patients were randomized into colostrum and placebo groups over an eight -week period (four weeks of treatment and four weeks of follow-up). By week four, symptom improvement was reported in 55.5% of the colostrum group versus 44.4% in the placebo group, though this difference was not statistically significant (Table 2). Symptom scores decreased modestly in both groups, and a slight decline in quality of life was observed. While results did not reach statistical significance due to the small sample size, they indicate a positive trend and support further investigation into the immunomodulatory and gut-protective effects of colostrum in IBS patients [97].

In another study conducted in this direction, probiotics were investigated for their role in regulating of local and systemic immune responses in both healthy individuals and individuals with IBS. In this study, a product containing *Lactobacillus* species, *larch arabinogalactan* and colostrum was supplemented for 22 days; blood and stool samples were taken from 20 IBS patients and 20 healthy individuals before and after the supplementation. After supplementation, the percentage of NK cells was assessed and found to be statistically lower in IBS patients compared to healthy subjects (*p* = 0.03). When stool analysis was performed, a decrease in IL-6, IFN-γ, TNF-α, and secretory IgA levels, and an increase in IL-10 and IL-17A levels were observed in IBS patients; but these changes were not statistically significant. A significant decrease in IL-6 and IFN-γ levels was recorded in healthy individuals whose feces were analyzed (*p* < 0.001). In addition to the findings of these analyses, improvements in clinical symptoms were observed in 65–75% of IBS patients and it was also reported that symptoms completely disappeared in 5 of 20 patients (Table 2). Lean colostrum used in this study increased the cytotoxicity of NK cells. In addition, it was emphasized that it supports mucosal immunity by providing GF, Ig, LF and various cytokines. Based on this study on the clinical use of colostrum, it can be concluded that various colostrum-containing formulations may provide regulatory effects on some immune parameters and improve clinical symptoms in IBS patients [98].

### 4.4. NSAID-Induced Enteropathy

NSAIDs are widely used in the treatment of musculoskeletal and chronic inflammatory disorders. However, long-term use of these drugs can result in clearly observable structural and functional changes in the GI tract, particularly in the small intestine. Mucosal erosions, ulcerative disease, and subepithelial hemorrhages have been found endoscopically in 70% of individuals exposed to long-term NSAID therapy. However, despite these findings, only 10% of these patients had dyspeptic symptoms. This may explain why lesions occurring in the small intestine are usually silent and difficult to detect. Acid suppressive agents such as proton bomb suppressors may be effective in the prevention of NSAID-induced diseases of the gastric mucosa. However, this method is not valid for NSAID-induced damage to the small intestine, and this has led to the need for alternative preventive strategies in the treatment of small intestinal diseases. Numerous studies in animal models have shown that BC is a potential protective agent against NSAID-induced intestinal damage. It is thought that the bioactive proteins contained in BC may be effective in mucosal barrier protection and may also have supportive effects in maintaining mechanical barrier integrity. Although the preclinical findings obtained from animal models are quite positive, the need for more comprehensive, long-term, and well-designed human studies is evident in order to learn to what extent this efficacy of BC is clinically effective in practice [115].

Chicken eggs and colostrum contain some proteins that regulate immune responses and exert growth GF effects. One study examined the effects of eggs on the GI tract, which have been less studied than colostrum in terms of their effects on intestinal defense and tissue repair, and observed the additional benefits of these effects when added to colostrum. The study groups were egg-only, colostrum-only and a combination of 40% egg and 60% colostrum and their effects on a number of different diseases, including NSAID-induced conditions were observed. The findings from the study showed that both eggs and colostrum increased cell proliferation and migration by 3-fold. To make a comment about BC based on these findings, it can be said that it has healing effects against diseases such as NSAID-induced enteropathy, where mucosal barrier integrity is impaired. The mice used in the study received the substances to be tested with drinking water for 7 days and were exposed to indomethacin at a dosage of 85 mg/kg subcutaneously on the day 7. Histological analysis evaluated villus height and morphologic changes. In this study, it was determined that BC exhibited protective effects against villus atrophy and provides positive effects with its bioactive components that protect mucosal integrity. Based on this information, it is evaluated that BC may have a protective effect against epithelial destruction occurring in NSAID-induced enteropathy. In another model in which the anti-inflammatory effects of BC were tested in pathologies similar to NSAID-induced enteropathy, the effects of egg and colostrum combinations were tested in Sprague Dawley rats after induction of colitis with DSS. In this model, histological damage score was evaluated according to MPO levels and this score was found to be lower in BC containing groups. Proliferative and migratory effects were investigated in vitro assays, and it was thought that colostrum containing EGF may also play a role in intestinal repair. The potential contribution of GFs in colostrum should be evaluated in cases where tissue integrity is impaired such as NSAID-induced enteropathy. In the indomethacin-induced villus shortening model, a significant protective effect was observed with colostrum administration alone. In addition, the fact that this protective effect was much higher in the egg and colostrum combination group indicates that these components have a synergistic protective effect when administered together. This effect of BC suggests that it can be used as a high potential supportive agent in complex intestinal inflammatory diseases such as NSAID-induced enteropathy [99].

The effects of milk and dairy products on morphological structures and EGF expression were investigated in a study on eighty male *Sprague Dawley* rats in order to evaluate small intestinal damage caused by NSAID use. The rats were randomly divided into five groups: control group, a diclofenac group, diclofenac group with 10% low fat milk, diclofenac group with 10% colostrum, and diclofenac group with yogurt. Milk, colostrum or yogurt applications were made with preclinical applications five days before diclofenac administration. After these applications, diclofenac was administered as a single dose of 15 mg/kg orally. Both anatomical and mucosal damage scores were evaluated in the analyses performed at 24 and 48 h after the applications. In addition, villus height was analyzed, mucosal structures were examined by transmission and scanning electron microscopy, and EGF levels were examined immunohistochemically. As a result of the electron microscopic evaluations, it was determined that the epithelial cell structures of the colostrum- supplemented group were better preserved and microvillus integrity was better preserved compared to the yogurt and milk supplemented groups. As a result of EGF expression evaluations, it was determined that colostrum showed certain protective effects, but milk and yogurt supplements were much less effective than colostrum in terms of this effect. Based on the results of this study, BC may have protective effects against NSAID-induced intestinal mucosal damage. The therapeutic potential of BC is supported especially in terms of its effects on villus height and preservation of epithelial integrity. It is suggested that these effects may be related to EGF and BC may directly contribute to mucosal barrier defense [100].

In another study evaluating the effects of BC supplementation in NSAID-induced diseases, findings on body weight, food and water intake, epithelial permeability and microbiota were evaluated. In this study, lower food, water, protein and calorie intake were observed in the diclofenac-treated groups compared to the control group. While there was no difference between the groups in most of these parameters, water intake was higher only in the diclofenac group given low-fat milk compared to the other diclofenac groups. In the determination of epithelial permeability in the groups, while the fractional excretion of orally administered ^51^Cr-EDTA was around 4% in the control group, diclofenac administration increased this rate by an average of six-to sevenfold and caused impaired intestinal permeability. In addition, simultaneous supplementation of BC suppressed the increase in fractional excretion and reduced the permeability rate by approximately fourfold compared to the control group. In this study, this effect on the epithelium was not observed in the skim milk treated group. The evaluation of the effect on the epithelium supported the positive effect of BC on the protection of mucosal barrier integrity. In the evaluation of the microbial load in the ileal lumen, the total number of Gram-negative bacteria in the control group was determined as 10^4^–10^7^ CFU/g. This number increased 100–10,000 times after diclofenac administration. On the other hand, in the BC-supplemented group, this increase was limited in the range of 20–500 fold. This shows the positive effects of BC on microbiota balance. On the other hand, milk supplementation did not show a significant effect in this regard. When serum total protein and albumin levels were evaluated, no decrease was detected in animals given diclofenac. This indicates that the results obtained were not related to liver function and that intestinal function was effective in the evaluations. When the findings obtained from this study are evaluated together, it is concluded that BC has protective effects on potentially vital problems such as increased intestinal barrier permeability caused by NSAIDs, disruption of microbiota balance, protein loss and has a therapeutic potential [101].

Long-term ingestion of NSAIDs was generally thought to have adverse effects on the upper GI tract, and therefore the damage to the small intestine was not investigated for a long time. However, new imaging techniques such as video capsule endoscopy have been developed and the damage caused by NSAIDs on the small intestine can be observed more clearly. Intestinal lesions caused by NSAIDs have become more visible with these imaging techniques, both in terms of their frequency and severity. In this context, a review evaluated the effects of complementary or alternative medicine (CAM) approaches on the small intestine. In this review, evidence from clinical and experimental studies examining the effects of CAM practices on NSAID-induced small intestinal injury was evaluated and a total of 22 studies (3 clinical, 19 experimental) were analyzed. These reviewed studies included 10 different CAM applications including BC, Orengedokuto, muscovite, licorice, wheat, brown seaweed, *Ganoderma lucidum* mushroom mycelium, Chaenomeles speciosa, and Jinghua Weikang capsule. Within the scope of the evaluations made with BC in this review, both experimental and clinical studies emphasize that BC may have different levels of protective effects against NSAID-induced intestinal damage. The mechanisms of these effects include regulation of intestinal permeability, protection of epithelial integrity, reduction of enteric bacterial load, and support of mucosal structure. Among the bioactive components of BC, especially GFs, Igs and nutritive proteins stand out as the components with the greatest effect on the prevention and repair of NSAID-induced mucosal damage. However, as stated in this review, it is still unclear whether these effects can maintain the same effect in long-term NSAID use. In the future, the duration of these effects, the effects of BC on NSAIDs and the therapeutic potential of BC on NSAID-induced enteropathy should be more clearly demonstrated and evaluated in larger-scale clinical studies [102].

Natural therapeutic agents such as Cordyceps sinensis and BC are gaining attention for their broad biological effects. BC contains Igs, antimicrobial proteins, GFs and nutrients, offering the potential for immune support and GI healing. In this study, the effects of BC and Cordyceps against NSAID-induced GI damage were evaluated in *Galleria mellonella larvae*. Larvae were fed diets with 10% colostrum, 10% *Cordyceps* or a combination of 5% + 5% before NSAID (indomethacin) or *Campylobacter jejuni* infection [104]. The 10% *Cordyceps* group showed the highest reduction in intestinal permeability and 77% survival. The BC group showed strong protection at the intestinal level but survival in systemic infection dropped to 50%. The combination group underperformed, possibly due to dose interactions. Larvae given colostrum gained more weight, which was attributed to the high protein and carbohydrate content of BC. On the other hand, the higher fat content of *Cordyceps* was associated with stronger effects on immune parameters. Systemic efficacy was limited; the lowest health indicators were observed in the BC group when the infection was delivered directly to the hemocoel. This suggests that the effect is more at the local level. The *G. mellonella* model is suitable for local effects; however, further studies in mammalian models are needed for systemic effects [103].

In a model examining the damage caused by NSAIDs to the GI tract and analyzing the effect of supplementation of LFs in BC on these damages, the protective effects of *Bifidobacterium longum* BB536 (2.5 × 10^6^ CFU/rat, twice daily) and LF (100 mg/kg, twice daily) against diclofenac-induced enteropathy in rats were investigated. Diclofenac was administered to rats at a dosage of 4 mg/kg per day for 14 days and caused significant histologic damage to the ileum tissue in these rats. This damage resulted in an increase in MPO and MDA levels and a significant increase in TLR-2, TLR-4, MyD88 and NF-κB p65 expression. In addition, fecal calprotectin levels increased, and blood hemoglobin levels decreased. In this model, when both LF and BB536 were administered alone, they suppressed the increase in MPO, MDA, NF-κB p65, and calprotectin and prevented the decrease in hemoglobin. No regulatory effect on TLR 4 was observed in LF treatment, whereas BB536 caused regulatory effects on TLR-4. The treatments had no effect on MyD88 expression, and the expression level remained high regardless of the treatments. A slight increase in TLR 2 expression was detected in all treatments (Table 2). When all treatments were considered, the highest protective effect was observed in the combined application of LF and BB536. There was a significant decrease in MPO levels and hemoglobin levels were close to normal. Based on the experimental findings of this study, it was determined that LF, an important component of BC, may have a direct effect on the reduction of mucosal damage due to NSAID-induced inflammation. At the same time, the fact that BC has ingredients similar to probiotic and prebiotic structure suggests that the synergistic protective effect determined in this study may also be provided by BC supplementation. These analyses suggest that BC may offer multifaceted support by targeting both immune receptors and oxidative stress markers in NSAID-associated intestinal damage [104].

NSAIDs are widely used due to their analgesic, antipyretic, and anti-inflammatory effects. Due to this widespread use, they have many side effects in the GI system, the most striking of which are peptic ulcer formation and intestinal tissue damage. In this study designed to evaluate this damage caused by NSAIDs, four different NSAIDs, indomethacin, diclofenac, aspirin, and ibuprofen, were used to experimentally induce gastropathy in mice. In the model of this study, the potential protective effects of this region against NSAID-induced mucosal damage were examined by isolating the C-terminal region of BC-derived LF. This serine protease-derived C-lobe fraction was able to reverse 47–70% of the damage to the intestinal mucosa when administered concomitantly with NSAIDs, whereas no significant effect was observed in the prevention of damage when administered simultaneously. Based on these results of the designed model, it was revealed that the LF C-lobe can bind to NSAIDs with high affinity and this affinity varies in the range of 2.6–4.8 × 10^−4^ M (as shown in Table 2). The results obtained from this study show that the C-lobe of LF, which is also found in BC, carries regions that are structurally highly compatible with NSAIDs and can be used as an effective protective agent in the prevention of gastropathies that may occur in long-term use of NSAIDs [105].

Another study based on the effect of NSAID-induced enteropathies emphasized the inadequacy of existing protective studies and the need for studies on new support strategies that specifically target intestinal barrier integrity. In the study conducted in this direction, the effects of BC, which stands out as a rich source of GFs, were observed against NSAID-induced increase in intestinal permeability in human experiments. In the first of 2 different models, healthy male subjects (*n* = 17) received either colostrum (125 mL, three doses per day) or whey protein (control) supplemented with 50 mg oral indomethacin 3 times a day for 5 days, and intestinal permeability was analyzed by lactulose/rhamnose protein control. The results of the analysis in the control group showed that indomethacin administration caused an increase in permeability (0.36 ± 0.07 → 1.17 ± 0.25; *p* = 0.01), while this increase in intestinal permeability was not observed in the BC group compared to the control group (Table 2). Based on these findings, the protective effects of BC on intestinal barrier integrity could be directly determined. In the second model, patients (*n* = 15) on high-dose and continuous NSAID treatment received BC or control solution supplementation for 7 days (125 mL, three times a day). In this group of patients, no significant improvement was observed as in the first study, as the initial permeability values were already low (0.13 ± 0.02). Based on this result, it can be inferred that the effect of BC is more significant especially in cases where permeability is impaired. A crossover design was used in both models, and a two-week washout period was applied between the interventions. The results obtained after these interventions showed that spray-dried and defatted BC preparations exhibited protective effects against NSAID-induced increase in intestinal permeability. Very strong evidence has been presented that BC, especially when administered in the acute phase, may reduce NSAID-induced GI damage by maintaining intestinal barrier integrity [106].

### 4.5. Necrotizing Enterocolitis (NEC) in Preterm Infants

Oropharyngeal colostrum supplementation (OAC) is emerging as a potential approach to prevent vital neonatal complications by supporting the immune system and controlling inflammation in premature neonates. One study investigated the potential effects of OAC on NEC and late-onset sepsis in premature infants with gestational age ≤ 32 weeks. This study was conducted in a tertiary neonatal intensive care unit in China and included a total of 252 infants randomized in a 1:1 ratio. 127 of these 252 infants were in the OAC group and 125 were in the control group. In this model, infants in the OAC group were supplemented with 0.4 mL of maternal colostrum every 3 h, starting within the first 48 h after birth. In the control group, saline was administered at the same dose and at the same time interval. According to the findings, the incidence of NEC (Bell’s stage 2 or 3) was 2.36% in the OAC group and 10.40% in the control group (Table 2). The relative risk was calculated as 0.23 (Cl 95%, 0.07–0.78) and similar values were observed with the correct analysis. These findings suggest that oropharyngeal colostrum administration may reduce the risk of neonatal infection by regulating the immune response and may be beneficial for the maturation of the GI tract. Based on this study, it can be concluded that BC-based applications have an important therapeutic potential in preventing NEC complications in premature infants due to similar content and functional properties. The rich bioactive content of BC suggests that the positive effects shown by OAC can be transferred to clinical practice through BC [107].

In an experimental study using a premature pig model, the effects on gut health and the effects against the development of NEC were analyzed by supplementing BC at different rates. Sixty-eight premature piglets at 90% gestational age, delivered by cesarean section, were divided into four groups that receiving increasing amounts of BC via the enteral route for eight days. Four groups were formed as BC00 (0% BC), BC25 with 25% BC, BC50 with 50% BC, and BC75 with 75% BC. All groups were received bolus feeding and analyzed by biochemical methods at the end of the fifth day. Although the frequency of mild NEC-like lesions was similar between the groups, a significant reduction in severe lesions was found in the BC75 group (27% vs. 79%; *p* < 0.05; BC00) (Table 2). These analyses suggest that high-dose BC administration may be effective in preventing NEC in NEC premature piglets. The results of this study suggest that BC may play a role in regulating the immune response by supporting mucosal integrity at high doses in premature individuals [108].

In another randomized controlled trial, the effects of oropharyngeal supplementation of colostrum on NEC in premature infants with very low birth weight (<1250 g) or less than 30 weeks of gestation were examined. In the design of the study, a total of 117 newborns were divided into two groups, 59 in the OAC group and 58 in the standard care group. OAC supplementation was initiated by administering 0.2 mL of colostrum from the mother in the first 24 h after birth and repeated every 2 h for the next 72 h, independent of enteral nutrition status. Regarding the results of the study, firstly, the incidence of NEC (stage 2 or 3) was lower in the OAC group compared to the standard care group, but this rate was not considered statistically significant. However, the average duration of hospitalization was seven days shorter in the OAC group. Although there was no statistically significant decrease in the frequency of NEC, the findings of this study suggest that OAC administration may have a reducing effect on recovery time in premature infants. It has been shown that the immunologic and bioactive components of BC may positively affect the clinical outcome by supporting the immune response, especially if administered oropharyngeally in the early postnatal period [109].

The effect of oropharyngeal administration of colostrum on morbidity and mortality in very preterm infants was evaluated in another randomized controlled study. A total of 260 preterm infants with gestational ages ranging from 26 to 31 weeks were included in this study. As the primary outcome, composite parameters including the development of NEC were evaluated. In the group with a mean gestational age of 29.5 weeks and a mean birth weight of 1201.7 g, these parameters were 33.6% in the colostrum group and 29.7% in the placebo group. This difference obtained as a result of this analysis was not considered statistically significant. In addition, no statistically significant results were obtained in the clinical parameters of NEC as a secondary outcome measure (Table 2). The findings of this study indicate that oropharyngeal administration of colostrum has no significant effect on reducing the incidence of serious clinical complications in this group of patients. However, since these results may be affected by various factors such as duration of administration, dose of administration, or timing of initiation, they should be reevaluated in larger groups and with different optimized protocols [110].

In another study using premature pigs, it was analyzed whether the negative effects caused by formula feeding in the first days after birth would be reduced by BC administered after formula feeding. The design of the experiment included a total of 74 premature piglets, which were fed either formula or BC in increasing amounts until Day 5. After this, the piglets were either euthanized or continued BC or formula until Day 9. A total of six groups were formed according to this design. These groups were BC only for five days (C5) or formula (F5); BC only for nine days (CC) or formula (FF); BC followed by formula (CF), and BC after formula (FC). By Day 9 of GI assessments, a similar decline in the incidence of NEC was observed in all six groups (15–21%) (Table 2). Based on the findings against NEC in this study, enteral supplementation with BC in the early postnatal period is thought to have significant positive effects on intestinal development and suppression of inflammatory responses. In addition, with these effects, BC provides a protective barrier against serious diseases such as NEC. These findings demonstrate that BC can be used as a potential therapeutic agent on NEC [111].

Another randomized, double-blind, placebo-controlled study investigated the effects of BC supplementation on the development of NEC and early sepsis in very low birth weight infants. A total of 86 premature infants were included in the study, with birth weight ≤1500 g, gestational age ≤32 weeks, and postnatal age ≤96 h. Participants were divided into two groups: one receiving BC via enteral administration and the other receiving placebo. The supplements were administered four times per day until the infants reached 21 days of age, with no interruption until discharge or death. The study results included outcomes such as definitively diagnosed NEC, sepsis, mortality, and IL-6 levels in stool. The results did not reveal any statistically significant differences between the two groups in terms of their effects on NEC. However, an increase in stool IL-6 levels was observed in the BC group, and radiological findings of ileus and NEC were reported more frequently in this group. These results indicate that BC does not provide the intended protective effects in this patient group and may even cause an increase in certain inflammatory response markers (IL-6). Based on the results of this study, BC supplementation does not show statistically significant effects in the prevention of NEC and has no clinical benefit. These findings suggest that the potential effects of early enteral supplementation in very low birth weight infants should be carefully evaluated in larger studies and that the effects of BC on this disease should be examined [112].

In a study, the effects of milk formulas enriched with BC, sialic acids, gangliosides, or osteopontin on GI function and NEC resistance in premature piglets were analyzed comparatively. A total of 47 premature piglets delivered by cesarean section were supported with parenteral nutrition for the first two days and then with enteral nutrition for 1.5 days. In this study, a diet containing BC was administered as a control formula (*n* = 4–6). The results of the study showed significant improvements in all intestinal function parameters (villus structure, enzyme activity, absorption capacity) in the groups fed with BC compared to the groups fed with formula milk. Protective effects against NEC complications were detected in the group fed the BC diet (Table 2). These effects of BC were not limited to the in vivo results of the study but were also confirmed in vitro. It was demonstrated that whey derived from BC modulates the cytokine response secreted by bacteria-stimulated mouse-derived DCs in a manner dependent on both the type of bacteria and the dose. This effect can be explained by the synergistic interactions of the bioactive components contained in BC. The results of this study attribute the multifaceted effects of BC in premature infants to the synergistic effect of the natural bioactive components in its composition and once again highlight its therapeutic potential against diseases such as NEC [113].

## 5. Formulations and Administration Routes

BC is available in various stabilized formulations such as powder, liquid, tablet, and capsule for oral administration. The main purpose of the development of these uses is to provide more feasible methods, while preserving the efficacy of the bioactive components of BC. In addition, randomized controlled clinical trials of tablet formulations containing HBC have shown significant protective effects against pathogenic microorganisms such as enterotoxigenic *Escherichia coli*. These effects provide important evidence supporting the usefulness of solid formulations of BC [116]. One of the most important points in determining formulations is the stability of the naturally rich bioactive content of BC. This is important because these components are delicate structures and can be easily denatured depending on the processing techniques applied. This lowers the bioavailability of the components. For this reason, the aim is to ensure both component stability and maintain their functional efficacy in the oral forms developed [11].

Dosing protocols in clinical applications of BC supplements vary according to the targeted age group and intended use. In the literature, there are randomized controlled trials evaluating various durations and dosing regimens in both children and adults. In a study conducted with preschool children, the administration of the BC preparation was carried out in two phases. BC was administered at a dose of 500 mg twice daily in the first 15 days, 500 mg once daily, and 500 mg once daily for the following 30 days. This application was prepared to reduce the frequency and severity of upper respiratory tract infections [117]. There is a wider dose range in the applications made in adults. These applications are usually administered at doses ranging from 10–60 g per day. This application is usually carried out in periods of 4 to 8 weeks and is used with the goals of supporting GI barrier functions, reducing exercise-induced intestinal permeability, and supporting the immune system [118]. For example, participants in healthy adult subjects were given 20 g of BC supplements daily for 2 weeks. These data suggest that BC can be used safely in clinical applications by determining the dosage range according to the target population. In addition to this information, differences in response between individuals and bioavailability levels that may vary depending on the formulation of the product used should also be considered.

The potential of BC to support GI health may be further enhanced when used in combination with probiotic and prebiotic agents. These synbiotic approaches aim to optimize the function of the intestinal barrier and regulate the inflammatory response by complementing the biological effects of BC. One study aimed to test the human tolerability of a combination of a probiotic strain of *Bifidobacterium infantis* and BCP, a BC product. The protocol of this study consisted of three phases, co-administration of the probiotic and prebiotic components during the first 5-week period, followed by a 2-week washout period, and the administration of the prebiotic supplement alone during the last 5 weeks. The results showed that the combination treatment was well tolerated. The most common side effect was mild flatulence [119].

The processing conditions to which the bioactive components of BC are exposed during the formulation process bring certain complications in both preserving biological activity and ensuring bioavailability. In studies comparing different processing techniques, a decrease in IgG levels was determined in all applications. However, this IgG loss was lower in BC products processed by freeze-drying compared to spray drying and pasteurization. It was observed that freeze-dried BC samples maintained a higher level of biological integrity in terms of both total protein content and defense proteins (IgG, IgA). These findings suggest that the stability of sensitive bioactive molecules, especially IgG, is directly dependent on the drying method applied. Exposure to high temperatures during the spray drying process leads to loss of bioactivity due to disruption of the structure of proteins. This not only shortens the shelf life of the product but also negatively affects its potential therapeutic effect by reducing absorption in the GI tract. Based on the findings of these studies, it is not only sufficient to select appropriate drying methods, but also to develop auxiliary systems to enhance targeted delivery and stability of these bioactive components. For this purpose, studies on liposomes, nanoemulsions, and other encapsulation technologies aim to ensure that the immunological components in BC are delivered to the target tissue while maintaining their functional activity.

## 6. Regulatory and Safety Considerations

BC has been included in GRAS (Generally Recognized as Safe) status in various food and beverage formulations, based on data provided by companies such as PanTheryx in the USA and expert panel evaluations [120]. When looking at the scope of this safe status, various toxicological tests have been applied on milk protein components in Europe. As a result of these tests, it was emphasized that components such as β-lactoglobulin, casein and LF, which are also found in BC, are safe in appropriate products, but the use of these products should be careful in individuals with potential milk protein allergy [119,121]. In clinical studies, BC is generally considered to be a well-tolerated supplement. The most common side effects that have occurred as a result of these uses are transient problems such as mild gas, short-term diarrhea, and abdominal discomfort. The results of BC supplementation in preterm infants have shown that BC supplementation is considered safe and does not cause milk protein allergy or lactose intolerance. Based on this information, more large-scale human studies are needed [122]. Particular attention should be paid to β-lactoglobulin and casein components in individuals with milk protein intolerance or milk allergy. In addition, the use of BC in these individuals should be supported by the evaluation of specialized formulations and low-allergen structures [121,123]. In addition, labeling and ingredient-lactose ratio should be determined in lactose intolerant individuals. If necessary, supplementation should be started with low tolerance doses [124].

## 7. Future Directions

The therapeutic potential of BC in various GI diseases is supported by current findings. In addition, there are many different research topics that need to be done in the future. Foremost among these research, large-scale randomized controlled trials are needed to evaluate the clinical efficacy and safety of BC supplementation against diseases such as IBD, IBS, NEC, and NSAID-induced enteropathy. The Ig, LF, GF and OS in BC need to be clearly defined. This is of great importance for the reproducibility of studies and regulatory approval. The development of new carrier systems, such as nanoencapsulation, to increase the bioavailability and efficacy of BC and its delivery to inflammatory target sites is promising for the consistency of the findings obtained in future studies. Furthermore, comparative studies with human milk-derived products can play an important role in revealing inter-species differences and possible translational benefits. Based on its effects on microbiota modulation, the synbiotic effects of BC in combination with probiotic and prebiotic agents can be evaluated. Thus, the effects of BC on microbiota composition associated with dysbiotic states can be investigated in more detail. Regulatory compliance and the long-term safety profile of BC are areas of research that still require large-scale studies. Issues such as cautious use in potentially allergenic individuals, quality control of commercial products, and long-term safety of use need to be elaborated from both clinical and regulatory perspectives. In this process, collaborations between clinical researchers, nutrition scientists, and regulatory agencies will ensure that the therapeutic potential of BC in GI health is fully realized. Issues such as careful use in potentially allergenic individuals, quality control of commercial products and long-term safety of use need to be elaborated from both clinical and regulatory perspectives. In this process, collaborations between clinical researchers, nutrition scientists and regulatory agencies will ensure that the therapeutic potential of BC in GI health is fully realized.

## 8. Conclusions

BC is a strong natural supplement that supports the health of the GI tract with its rich bioactive ingredients. Due to its components such as Igs, LFs, GFs, OSs, and bioactive peptides, BC has the ability to modulate immune responses, protect epithelial integrity, reduce intestinal permeability, and suppress inflammatory processes associated with dysbiosis. The effects of BC on the GI tract against diseases such as IBD, IBS, NSAID-induced enteropathy, NEC have been demonstrated both in animal models and in data obtained in a limited number of clinical studies. Findings from these studies have revealed that BC may have therapeutic effects in different pathophysiological conditions. However, standardization of formulations, long-term safety data, and large-scale randomized clinical trials are required for these positive effects to be more widely accepted in clinical practice. Moreover, further studies should demonstrate which bioactive components or combinations of components make BC more effective. The mechanisms presented in this review and the scientific evidence demonstrated by clinical trials suggest that BC is a potential complementary therapy candidate for the prevention and management of GI diseases. The need for multidisciplinary and translational research to improve the existing scientific base and expand its clinical application areas remains. While the review by [123], mainly focuses on the compositional characterization and nutritional value of BC and its general applications on human health, it provides limited discussion on molecular mechanisms and translational perspectives. In contrast, the present review expands upon these foundations by integrating recent mechanistic insights and translational evidence published after 2019. Specifically, we discuss the therapeutic potential of BC and implications for GI health. The emerging challenges are associated with standardization, formulation, and large-scale clinical application.

## Figures and Tables

**Table 1:** Major Bioactive Components of BC: Concentrations and Functions.

Growth Factors	Concentration in BC (ng/L)	Functions	References
EGF	324.2	EGF stimulates cell proliferation, initiates signal transduction via binding to an EGF receptor and promotes DNA repair and replication.	[16,17]
TGF-β2	1130.43	TGF-β1 elevates cell proliferation, modulates cell migration (colostrum effect on cell migration decreased with the addition of TGF-β neutralizing antibody). Moreover, it is essential for mucosal immunity	[18,19]
IGF-1	267.97	IGF-1 contributes to cell proliferation in GI cells and is tolerant to 40–60 °C heat conditions. IGF-1 is involved in GI tissue repair, increased permeability, and cell repair mechanisms.	[19]
TGF-α	200	TGF-α contributes to the repair of the intestinal mucosa, has an intestinal affinity similar to EGFR, and has an intestinal barrier function	[18,19]
Immunoglobulins	Concentration in BC (g/L)	Functions	References
IgG_1_	44.96	IgG1 is the major immunoglobulin isotype and plays a major role in the gaining of passive immunity, neutralizing enteric pathogens.	[20]
IgG_2_	2444	IgG_2_ is essential for the immune responses and passive immunity transfer.	[20]
IgA	1–5	IgA has properties such as regulation of gut microbiota, mucosal immune balance, and bacterial infection protection.	[21,22]
IgM	5.07	IgM plays a key role in mucosal immunity, immune response, and neutralizing pathogens	[22,23]
Total IgG	30–87		
Lactoferrin (LF)	Concentration in BC (g/L)	Functions	References
Total LF	1–5	LFs are iron binding proteins. These proteins have several beneficial effects on an organism’s health; they have antibacterial, antifungal, antiviral, antiparasitic, anticancer, and immunomodulatory properties. Proliferation of cells called osteoblasts is considered to have positive effects on Parkinson’s disease through reducing oxidative damage, affecting the blood-brain barrier	[24,25]
Glycans	Concentration in BC (g/L)	Functions	References
3′ sialyl lactose	0.867 g/L	They have prebiotic functions; they are involved in the growth of beneficial bifidobacterium. 3′-Siallyactose, 6′-siallyactose are involved in nervous system development, myelization, and learning processes. Glycans also have a key function in the regulation of the gut microbiota.	[26,27]
6′ sialyl lactose	0.136 g/L		
6′ siayllactosamine	0.220 g/L		
disialyllactose (DSL)	0.283 g/L		
Total Glycans			

**Table 2:** Supplementation of BC components on GI disorders.

Disorder	Study Design and Sample Size	Dose Duration	Population Outcome	References
Diarrhea in preweaning dairy calves	Randomized controlled trial with 3 treatment arms (CON, STC, LTC) *n* = 108 preweaning calves)	8 feedings over 4 days: 2.5 L of 50:50 milk replacer and colostrum replacer (65 g/L each)	LTC group had faster diarrhea resolution and 98 g/day higher weight gain over 56 days compared to control	[79]
Childhood infectious diarrhea	Meta-analysis of 5 RCTs	Not specified	BC reduces stool frequency by 1.42/day, diarrhea occurrence by 71%, and pathogen positivity (OR = 0.29)	[80]
Calf diarrhea (BRV, BCoV, ETEC, Crypto)	Meta-analysis (41 studies, 94 sub-studies)	Not specified	Highest pooled prevalence: BRV-Crypto (6.69%); diagnostic method influenced detection	[81]
Infant diarrhea and RTIs	Multi-center, randomized, blank-controlled intervention trial, *n* = 192 term infants, (96 intervention, 96 control group)	1 sachet/day 3 months	BC reduced diarrhea incidence (RR = 0.25), duration, appetite loss, and respiratory symptoms	[82]
Shigellosis (children, *S. dysenteriae* type 1)	RCT: HBC vs. BC + antibiotic, *n* = 69 children (34 HBC group, 35 control group)	100 mL HBC × 3/day for 3 days	No significant difference in symptoms; stool culture positivity: HBC 6% ve BC 14%	[83]
Infectious diarrhea and SIBO (in vitro)	Caco-2 cell monolayer study, In vitro study, no animal or human subject	Not applicable (in vitro study)	BC ± egg protected barrier function, reduced apoptosis, preserved tight junctions	[84]
EPEC diarrhea (infants	Double-blind, randomized field trial, *n* = 125 infants (107 with complete data)	Supplemented for 7 days	BC Ig-supplemented formula lowered diarrhea incidence and duration; better weight gain	[85]
Shigellosis (*S. flexneri* macaque model)	In vivo challenge model with Travelan^®^, *n* = 12 (8 Travelan^®^ group, 4 placebo group)	Travelan^®^ orally, twice daily for 6 days	75% protection post-challenge in HBC group	[86]
Neonatal calf diarrhea (BRV, BCoV)	Cross-sectional observational study, *n* = 110 neonatal calves (<30 days old) from 57 daily herds	Colostrum timing within 12–24 h of birth (feeding timing analyzed)	BRV: 3.64%, BCoV:0.91%; BRV associated with sex and feeding time	[87]
Cryptosporidiosis (neonatal calves)	Observational (IgG quantification); *n* = 50 dam-calf pairs (50 dams and their newborn calves)	Colostrum collected <12 h after birth; IgG 570–4070 mg/dL	Higher anti-C. parvum IgG in colostrum associated with reduced infection (r = −0.425)	[88]
Ulcerative colitis (acetic acid-induced)	In vitro (rat mode, 4 groups); *n* = 37 Sprague-Dawley rats	300 mg/kg BC (oral or rectal), 7 days	Reduced weight loss, increased SOD levels, decreased CRP, WBC and histopathological damage	[89]
Distal colitis	RCT, double-blind, *n* = 14 patients with mild to moderate distal colitis (colostrum group 8, placebo group 6)	100 mL of 10% BC enema twice daily for 4 weeks	Symptom score decreased by −2.9 in colostrum group versus +0.5 in placebo, histological improvement in 5 of 8 patients with colostrum	[90]
TNBS-induced colitis	In vivo study; *n* = 24 mice (BC group = 12, control group = 12)	7 days BC pre-treatment before TNBS	Body weight loss was reduced; expression levels of TLR4, IL-1β, IL-8 and IL-10 were lower; beneficial bacteria population were higher in colostrum group	[91]
TNBS-induced colitis	In vivo study; *n* = 24 mice (BC group = 12, control group = 12)	300 mg/kg BC for 21 days before TNBS	Body weight loss and histological damage were reduced; TLR4, IL-1β, IL-8 and IL-10 expression was lower; microbiota changes were prevented	[92]
DSS-induced colitis	In vivo study; *n* = not explicitly stated mouse model	200 mg/kg BC daily for 2 weeks	Colitis severity was reduced based on body weight and colon length; inflammation was reduced; changes in immune cell populations were observed	[13]
DSS and T cell transfer colitis	In vivo study; *n* = not explicitly stated, mouse model	100 mg/kg IMM-124E (colostrum-based) daily by oral gavage	Mucosal damage was less severe, with reduced effector, T cells and increased regulatory T cells; systemic LPS exposure was decreased	[93]
DSS induced colitis (dose-response)	In vivo study; *n* = not explicitly specified, DSS induced mixed colitis model in mice treated with 4 different BC dose levels	100–200 mg/kg BC daily	Disease activity index and histological damage were reduced; tight junction proteins and microbiota diversity were improved; Akkermansia increased, *Escherichia-Shigella* decreased	[94]
Inflammatory cell stimulation	In vitro study using human intestinal epithelial cell lines (Caco-2 and HT29 cells)	Dose-dependent concentrations of colostrum	IL-8 levels decreased after TNF-α or AIEC stimulation; bacterial adherence to cells was reduced; direct antimicrobial effect observed	[95]
Diarrhea-predominant IBS (IBS-D)	Randomized, double-blind, placebo-controlled clinical trials, 30 patients with diarrhea-predominant IBS (SBI group = 15, placebo group = 15)	5 g/day or 10 g/day serum-derived bovine Ig (SBI) for 6 weeks	10 g/day group showed reductions in abdominal pain, loose stools, bloating, flatulence, urgency, and overall symptoms; 5 g/day group showed reductions in the symptoms	[96]
IBS (mixed types)	Prospective, double-blind randomized, placebo-controlled clinical trial, *n* = 18 patients with IBS (BC group = 9, placebo group = 9)	15 mL/day oral BC (MuKoBa™) for 4 weeks + 4-week follow up	No significant differences in symptom scores, quality of life, or endotoxin levels between groups; slight improvement trends observed in BC group	[97]
IBS	Single-center, blinded trial *n* = 40 participants (20 patients with IBS, 20 healthy individuals	21-day supplementation with combined product (*Lactobacillus* spp., *larch arabinogalactan*, and colostrum)	Clinical improvement in 65–75% of IBS patients, complete resolution in 5/20; decrease in pro-inflammatory markers and increase in IL-10 and IL-17A; changes not statistically significant in IBS group	[98]
Indomethacin-induced intestinal injury	In vitro (Caco-2, AGS, RIE-2), In vivo = 4 groups of adult mice In vivo = 3 groups of Sprague-Dawley rats	20 mg/kg/day for 7–9 (oral); 1 mg/mL in vitro	Colostrum and egg increased cell proliferation and migration; reduced villus shortening and colonic damage; combination more effective than either alone	[99]
Diclofenac-induced small intestinal damage	In vivo; *n* = 80 male Sprague-Dawley rats, divided into 5 groups	Diclofenac (15 mg/kg once); 10% colostrum orally for 5 days prior	Colostrum group had lower lesion scores and mucosal damage higher villus height and EGF expression vs. diclofenac; milk and yogurt showed no significant effect	[100]
Diclofenac-induced small intestinal injury	In vivo; *n* = 4 animal groups (number per group not specified)	Dİclofenac (100 mg/kg once); colostrum for 5 days before	Colostrum reduced intestinal permeability, enteric bacterial overgrowth, protein loss, and villus damage compared to diclofenac alone; milk was not effective	[101]
NSAID-induced small intestinal injury	Systematic review (22 studies: 3 clinical, 19 experimental)	Various models and regimens (review data)	Colostrum listed among CAMs that reduce permeability; bacteria, cytokines, and improve repair; mechanisms include prostaglandin increase, oxidative stress reduction	[102]
Indomethacin-induced gut leakiness	In vivo, *n* = *Galleria melonella* larvae (exact number not specified) divided into 4 groups	10% (*w*/*w*) colostrum in feed	Colostrum-fed insects showed resistance to indomethacin-induced gut leakiness; better survival compared to standard diet	[103]
Diclofenac-induced enteropathy	In vivo (rat); *n* = 40-week-old male rats (exact number not specified) divided into 4 groups inflammation, TLR, oxidative stress	LF 100 mg/kg, twice daily, 14 days	LF and *Bifidobacterium* reduced MPO, MDA, NF-κB p65, calprotectin; LF preserved hemoglobin, combination gave additional benefit	[104]
NSAID-induced gastric/intestinal injury	In vivo (mouse); structural binding studies, *n* = Mouse models treated with four NSAIDs; exact number of groups not specified	C-lobe of LF; 4 NSAID tested	Co-administration of LF C-lobe prevented 47–70% of NSAID-induced injury; x-ray structure revealed NSAID binding site on C-lobe	[105]
Indomethacin-induced gut permeability	Randomized crossover trial in humans, *n* = 7 healthy male volunteers (randomized crossover trial) and 15 patients on long term NSAID therapy	125 mL colostrum three times daily for 5–7 days	In volunteers, colostrum prevented 3-fold permeability increase caused by indomethacin; no effect in patients on long-term NSAIDs	[106]
NEC and late-onset sepsis	Pilot, single-center, parallel RCT, *n* = 252 preterm infants (OAC group = 127, control group = 125) with gestational age <32 weeks	0.4 mL maternal colostrum oropharyngeallyally every 3 h for 10 days	The incidence of NEC was lower in the OAC group (2.36% vs. 10.40%; 4.72% vs. 13.60%)	[107]
NEC and intestinal immaturity	Preclinical, randomized piglet study, *n* = 68 preterm piglets (90% gestation), divided into 4 groups	8 daily bolus feedings with 0%, 25%, 50%, or 75% BC for 5 days	BC75 reduced severe NEC-like lesions (27%, vs. 79% in BC100), improved gut permeability	[108]
NEC in very-low-birth-weight infants	RCT with 117 infants (<1250 g or <30 weeks)	0.2 mL maternal colostrum every 2 h for 72 h	There was no significant reduction in the incidence of NEC (0% vs. 7.1%) but hospital stay was shorter (34.2 vs. 41.5 days	[109]
NEC, LOS, and death in very preterm infants	RCT with 260 infants (26–31 weeks GA)	0.2 human milk or placebo every 3 h until oral feeds started	The composite outcome (death, NEC, LOS) was not significantly different between groups (33.6%, 29.7%).	[110]
NEC, diarrhea, and intestinal inflammation	Preclinical, randomized piglet study, *n* = 74 preterm piglets divided into six feeding groups	BC or F for 5 days, then maintained or switched for 4 more days	BC feeding decreased NEC (27% vs. 64%), diarrhea (16% vs. 49%), and improved immunity and intestinal function	[111]
NEC and sepsis in VLBW infants	Randomized, double-blind, placebo-controlled pilot trial, *n* = 86 very-low-birth-weight infants (BC group = 43, placebo group = 43)	BC or placebo 4× daily until day 21, discharge, or death	No clinical benefit was detected; trends towards increased IL-6 and NEC features in colostrum group	[112]
NEC and impaired gut function	Preclinical piglet study with control and enriched formula compounds, *n* = 47 preterm piglets delivered by caesarean section	BC, SL, Gang, or OPN enriched formulas fed over 1.5 days after 2 days TPN	All intestinal parameters significantly improved in pigs fed BC vs. formula; SL and Gang were ineffective	[113]

## Data Availability

No new data were created or analyzed in this study. Data sharing is not applicable to this article.
